# Methodological challenges in systematic reviews of mHealth interventions: Survey and consensus-based recommendations

**DOI:** 10.1016/j.ijmedinf.2024.105345

**Published:** 2024-01-29

**Authors:** Jesus Lopez-Alcalde, L. Susan Wieland, Jürgen Barth, Rebecca Grainger, Nancy Baxter, Neil Heron, Andreas Triantafyllidis, Carme Carrion, Eleonora M.C. Trecca, Felix Holl, Ana Maria Wägner, Sarah Edney, Yuqian Yan, Concepción Campos-Asensio, Gemma Villanueva, Rachelle R. Ramsey, Claudia M. Witt

**Affiliations:** a Institute for Complementary and Integrative Medicine, University Hospital Zurich and University of Zurich, Zurich, Switzerland; b Faculty of Medicine, Universidad Francisco de Vitoria (UFV), Madrid, Spain; c Instituto Ramón y Cajal de Investigación Sanitaria (IRYCIS), Unidad de bioestadística clínica, Hospital Universitario Ramón y Cajal, (CIBERESP), Madrid, Spain; d Center for Integrative Medicine, University of Maryland School of Medicine, Baltimore, MD, United States; e Department of Medicine, University of Otago Wellington, New Zealand; f Melbourne School of Population and Global Health, University of Melbourne, Melbourne, Victoria, Australia; g Centre for Public Health, Queen‘s University Belfast, Northern Ireland, School of Medicine, Keele University, Staffordshire, England, United Kingdom; h Information Technologies Institute, Centre for Research and Technology Hellas, Thessaloniki, Greece; i eHealth Lab Research Group, Universitat Oberta de Catalunya (UOC), Spain; j Department of Otorhinolaryngology and Maxillofacial Surgery, IRCCS Hospital Casa Sollievo della Sofferenza, San Giovanni Rotondo (FG), Italy; k Department of Otorhinolaryngology, University Hospital of Foggia, Foggia, Italy; l DigiHealth Institute, Neu-Ulm University of Applied Sciences, Neu-Ulm, Germany; m Institute for Medical Information Processing, Biometry, and Epidemiology, Ludwig Maximilian University of Munich, Munich, Germany; n Endocrinology and Nutrition Department, Complejo Hospitalario Universitario Insular Materno-Infantil, Instituto de Investigaciones Biomédicas y Sanitarias, Universidad de Las Palmas de Gran Canaria, Las Palmas de Gran Canaria, Spain; o Saw Swee Hock School of Public Health, National University of Singapore, Singapore; p Biblioteca Médica, Hospital Universitario de Getafe, Madrid, Spain; q Cochrane Response, Cochrane, London, UK; r Division of Behavioral Medicine and Clinical Psychology, Cincinnati Children’s Hospital Medical Center, Cincinnati, OH, United States; s Department of Pediatrics, University of Cincinnati College of Medicine, Cincinnati, OH, United States; t Institute for Social Medicine, Epidemiology and Health Economics, Charité – Universitätsmedizin Berlin, Berlin, Germany

**Keywords:** Digital health, eHealth, Systematic reviews, Meta-analysis, Survey

## Abstract

**Objective:**

Mobile Health (mHealth) refers to using mobile devices to support health. This study aimed to identify specific methodological challenges in systematic reviews (SRs) of mHealth interventions and to develop guidance for addressing selected challenges.

**Study Design and Setting:**

Two-phase participatory research project. First, we sent an online survey to corresponding authors of SRs of mHealth interventions. On a five-category scale, survey respondents rated how challenging they found 24 methodological aspects in SRs of mHealth interventions compared to non-mHealth intervention SRs. Second, a subset of survey respondents participated in an online workshop to discuss recommendations to address the most challenging methodological aspects identified in the survey. Finally, consensus-based recommendations were developed based on the workshop discussion and subsequent interaction via email with the workshop participants and two external mHealth SR authors.

**Results:**

We contacted 953 corresponding authors of mHealth intervention SRs, of whom 50 (5 %) completed the survey. All the respondents identified at least one methodological aspect as more or much more challenging in mHealth intervention SRs than in non-mHealth SRs. A median of 11 (IQR 7.25–15) out of 24 aspects (46 %) were rated as more or much more challenging. Those most frequently reported were: defining intervention intensity and components (85 %), extracting mHealth intervention details (71 %), dealing with dynamic research with evolving interventions (70 %), assessing intervention integrity (69 %), defining the intervention (66 %) and maintaining an updated review (65 %). Eleven survey respondents participated in the workshop (five had authored more than three mHealth SRs). Eighteen consensus-based recommendations were developed to address issues related to mHealth intervention integrity and to keep mHealth SRs up to date.

**Conclusion:**

mHealth SRs present specific methodological challenges compared to non-mHealth interventions, particularly related to intervention integrity and keeping SRs current. Our recommendations for addressing these challenges can improve mHealth SRs.

## 1. Introduction

Mobile Health (mHealth) refers to using mobile devices, such as smartphones, to support medical and public health practices [1–3]. mHealth can empower patients, carers, healthcare professionals and the general population [3–7] by improving health behaviour and adherence to treatment or by delivering interventions (*e.g.*, psychotherapy).

mHealth is a fast-developing field: there are over 350,000 health applications (apps), and there is an exponential increase in mHealth studies [7–10]. Although mHealth apps are advertised as improving health and well-being, systematic reviews (SRs) of randomized trials (RCTs) are needed to acquire robust evidence of their effectiveness [11]. SRs of mHealth interventions (hereinafter mHealth SRs) are also common [12]. Still, they may have different methodological challenges than SRs of conventional interventions (hereinafter non-mHealth SRs), such as medications. First, drug evaluation methods are only partially transferable to mHealth research. Second, apps can be developed and updated faster than drugs, which challenges SRs to remain current. Third, the evaluation and reporting of mHealth intervention integrity (the degree to which the intervention was implemented as intended) in clinical trials vary. This poses difficulties in SRs because it can lead to biased estimates of intervention effects and limit the ability of SRs to provide reliable evidence [13].

Identifying and overcoming the methodological challenges specific to mHealth SRs is critical to understand the effects of mHealth and, thus, to determine if mHealth can improve health outcomes [7]. This article has two aims: 1) To identify specific methodological challenges in SRs evaluating the effects of mHealth interventions; 2) To develop guidance to address selected methodological challenges.

## 2. Materials and methods

**Fig. 1.**
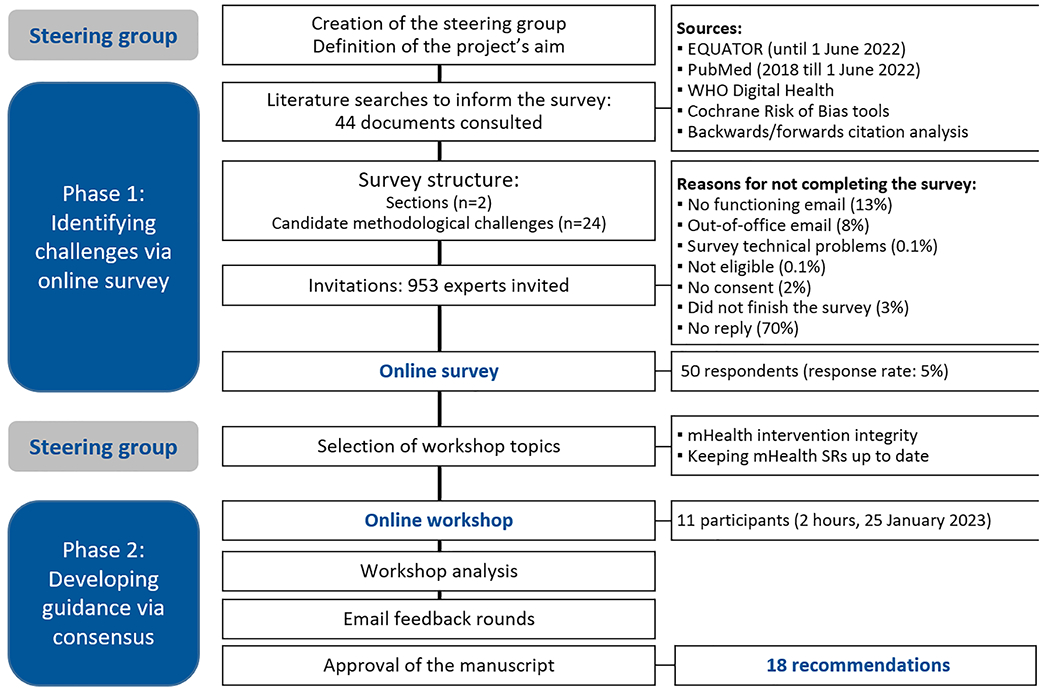
Study flow.

We performed a two-phase participatory research project with quantitative and qualitative methods (Fig. 1).

The steering group (SG) defined the project’s aims, collected potential methodological challenges of mHealth SRs, developed the survey, identified the survey respondents, and selected them for the workshop based on availability. The SG also analyzed the survey results, selected the topics for the workshop, summarized participants’ comments during the meeting, and integrated their feedback to generate this manuscript.

Phase 1. Identifying methodological challenges specific to mHealth SRs

Study design: cross-sectional web-based survey.

### 2.1. Survey items

The survey aimed to identify methodological challenges specific to mHealth SRs compared to non-mHealth SRs. We performed non-systematic searches in relevant sources until 1 June 2022 looking for methodological challenges potentially relevant in mHealth SRs (Fig. 1; Appendix 1). The SG chose methodological aspects potentially specific to mHealth SRs based on consensus (complete list available upon request). We created an anonymous online survey with *soSci Survey*. The SG and two external researchers piloted the survey, which was available in English for one month from 11 October 2022 (Appendix 2). The first survey section listed 24 potential methodological challenges in four groups according to the SR process. Participants were asked how challenging they found each methodological aspect in mHealth SRs compared to non-mHealth SRs. The response options were ranked on a five-category scale: “much less challenging”, “less challenging”, “similar challenges”, “more challenging”, or “much more challenging”. The “I don’t know” option was available. Participants could comment/propose additional challenges. The second survey section characterized the researchers’ academic background and SR experience. Finally, the survey invited the respondents to the online workshop (participation was optional, and responses were stored separately).

### 2.2. Survey sample

953 corresponding authors of mHealth SRs (Web of Science; 1 January 2018 to 17 June 2022) were invited by email to complete the survey. A librarian designed the search strategy for identifying mHealth SRs and obtaining the corresponding authors’ contact details (Fig. 1, Appendix 3). Only authors of at least one mHealth SR and one non-mHealth SR were eligible (the survey asked to confirm this requirement).

### 2.3. Survey analysis

Survey data were summarised using descriptive statistics (percentages for categorical variables, means and standard deviations (sd) and medians and interquartile ranges (IQR) for quantitative variables) in narrative and tabular formats. Statistical analysis was performed with R software [14]. We report the proportion of participants perceiving each methodological aspect as more or much more challenging for mHealth compared to non-mHealth SRs. Results are presented for the whole sample and stratified according to respondent experience (experienced respondents authored at least two mHealth SRs). We implemented thematic analysis to group participants’ comments into overarching categories [15].

Phase 2. Developing guidance to address methodological challenges in mHealth SRs

### 2.4. Online workshop

We organized a two-hour online workshop with SR authors, which was recorded with the participants’ consent. We invited the 37 survey respondents that showed interested in participating and six additional experts (Cochrane Iberoamerica and Cochrane Response). The workshop aimed to develop recommendations to address methodological challenges specific to mHealth SRs. The SG chose two topics for the workshop (mHealth intervention integrity and keeping mHealth SRs up to date) for the following reasons. Firstly, these topics were often rated as more or much more challenging by experienced systematic reviewers in the survey. For instance, 88 % of these reviewers identified the task of keeping mHealth SRs current as more or much more challenging. Additionally, aspects related to the integrity of mHealth interventions, especially those associated with data extraction, were deemed more or much more challenging by up to 80 % of the experienced reviewers. Secondly, the ongoing need to update apps is intrinsically linked to the integrity of mHealth interventions. Thirdly, the topic of mHealth intervention integrity was in alignment with a concurrent workshop that addressed methodological challenges in mHealth RCTs (details of which have been submitted for publication elsewhere). Recommendations from the workshop were endorsed by consensus (Appendix 4: workshop slides).

### 2.5. Workshop analysis and feedback rounds

The SG performed a thematic analysis to summarise the workshop discussions [15]. The survey results and the workshop recommendations were integrated into the first manuscript draft, which was emailed to the workshop participants. Moreover, two external mHealth SR authors commented on the manuscript. The SG incorporated their feedback into the final version of the manuscript.

## 3. Results

### 3.1. Survey results

We identified 1073 mHealth SRs and 953 corresponding authors’ mails. Fifty mHealth SR authors (50/953; 5 %) completed the survey. One additional author completed the survey but was excluded because he had authored mHealth scoping reviews. Fig. 1 details the reasons for not completing the survey. The most frequent academic backgrounds among the respondents were psychology (28 %), medicine (28 %), and epidemiology/public health (26 %). Half the participants (n = 25) were experienced SR authors. The respondents had authored a median of two mHealth SRs (IQR 1–3) and five non-mHealth SRs (IQR: 2.25–5) (Table 1).

All respondents identified at least one methodological aspect as more or much more challenging in mHealth SRs. Survey respondents rated a median of 11 (IQR 7.25–15) out of 24 aspects (46 %) as more or much more challenging (Appendix 5: Survey data set). The methodological aspects most frequently perceived as more or much more challenging in mHealth SRs were: defining the intervention intensity and components (85 %), extracting the mHealth intervention details (71 %), dealing with dynamic research with continuously evolving interventions (70 %), assessing intervention integrity (69 %), defining the intervention at the protocol stage (66 %), maintaining an updated review (65 %), defining the comparator (63 %), and dealing with co-interventions (60 %). Defining the population and identifying the design of the retrieved studies were the aspects described by the lowest number of respondents as more or much more challenging (12 % and 32 %, respectively) (Table 2).

Experienced and non-experienced authors’ perceptions were generally similar. However, more experienced authors considered the following aspects as more or much more challenging: maintaining an updated review (88 % versus 42 %), considering preprints (53 % versus 11 %), and managing studies with a large amount of missing data (56 % versus 32 %).

### 3.2. Online workshop

**Fig. 2.**
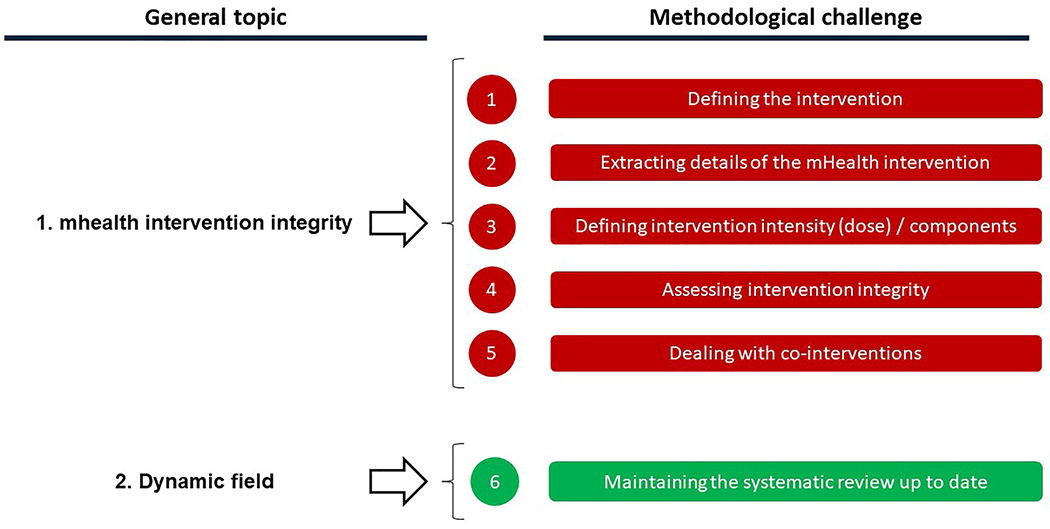
Workshop discussion points.

Eleven SR authors attended the online workshop (Table 3). Fig. 2 outlines workshop topics.Table 4.

### 3.3. Recommendations for addressing methodological challenges specific to mHealth SRs

The following recommendations were made with consensus agreement during the workshop, with refinement by email, after the workshop (recommendations explained in Appendix 6).

#### 3.3.1. Definition of the eligible mHealth intervention

##### Recommendation 1. Consider performing/consulting a scoping review to inform the protocol of the mHealth SR.

Scoping reviews providing an overview of previous SRs and relevant studies can help define the review question, identify evidence gaps, provide recommendations for future research, or identify strengths and limitations of available research [16]. We recommend following recent scoping review guidance, such as the Joanna Briggs Institute (JBI) Manual for Evidence Synthesis [17], in order to assess the appropriateness of the scoping review, the extraction, analysis, and presentation of the results, as well as the implications for clinical practice and research.

##### Recommendation 2. The SR eligibility criteria should clearly describe the eligible mHealth intervention, including details of the app.

SR authors should define clear inclusion criteria and predetermine how to deal with poor reporting in primary studies. As part of the inclusion criteria, authors should specify the minimum characteristics an app should have to be eligible (see Box 1). There is a need to standardize and develop a comprehensive mHealth app assessment tool beyond MARS and ABACUS to assist in this [18–20]. The protocol should also state whether studies of no longer functioning apps or operating systems are eligible and if they will be combined with studies of working apps or examined separately.

##### Recommendation 3. mHealth intervention SRs should define the technical context in which the review findings will be applied.

Considering the technical context in the eligibility criteria can help ensure relevant and feasible review findings. For example, mHealth interventions requiring high-speed internet may not be feasible in areas with limited infrastructure.

##### Recommendation 4. Do not neglect low-cost and low-tech Health interventions by default.

Including low-cost and low-tech mHealth interventions in SRs can help ensure that the solutions are feasible and sustainable for different healthcare settings, regardless of their infrastructure. For example, static text message-based intervention and a dynamic mobile app intervention may be included.

#### 3.3.2. Search methods

##### Recommendation 5. Develop validated filters for search strategies of mHealth interventions and use automation screening tools.

Search strategies using a filter for identifying mHealth intervention studies would improve the efficiency of the search process by reducing the number of irrelevant studies to screen [21]. Search filters to find articles on mHealth interventions should be developed and validated according to rigorous methods, such as appraisal checklists [22]. Consider also using SR software with automation tools for increasing screening efficiency, such as EPPI-Reviewer, DistillerSR, Covidence or Rayyan.

##### Recommendation 6. Carefully select the sources to search in mHealth SRs.

Search methods guidance in mHealth SRs is needed to address specific challenges. Examples are diverse evidence dissemination channels, heterogeneous terminology, rapidly evolving field, and irrelevant sources due to the low quality of the studies. Also, mHealth SRs have diverse information needs to support the review process, such as information on the intervention technology, outcomes, or implementation. SR authors should involve a specialized librarian to choose the most relevant sources.

#### 3.3.3. Data extraction

##### 3.3.3.1. Extracting the mHealth intervention details. Recommendation 7. SR authors should follow standardized data extraction and description of mHealth apps. Available reporting guidelines can help.

Standardized templates can guide data extraction and accelerate SR processes. Available reporting guidelines for primary studies, such as TIDieR-telehealth [23], can help SR authors design their extraction forms. However, the minimal list of characteristics to extract from each mHealth intervention must be agreed upon. Box 1 suggests features of the mHealth intervention, but SR authors should design and pilot-test specific forms to ensure they include all relevant details.

##### Recommendation 8. The review team should schedule time and training to extract the mHealth intervention characteristics.

Setting aside adequate time for training, piloting, data extraction and reaching consensus is essential due to the complexity of mHealth intervention data and the diverse information sources. Consider using tools for estimating how long the review will take to complete, such as PredicTER (Predicting Time requirements for Evidence Reviews) [24].

##### Recommendation 9. Make data extraction forms publicly available.

SR authors should make their data extraction forms publicly available and be explicit about any modifications made to established forms. This will save time for other SR authors since developing and piloting extraction forms in mHealth SRs is time-consuming. Consider making data extraction forms available in open repositories such as Figshare, Open Science Framework or Zenodo.

##### Recommendation 10. Data extraction of mHealth interventions should consider at least two levels: the intervention itself and the app specifications.

The review should define whether the evaluation will focus on the app and its components or the mHealth intervention outcomes. Thus, data extraction should consider at least two levels: the complex intervention (target population, intervention components, and outcomes); and the app itself (features and version, functionality, availability, and country of use).

##### Recommendation 11. Repositories collecting certification bodies’ decisions on mHealth apps can help SR authors.

Certification bodies can have useful information for authors of mHealth SRs. However, the certification criteria are heterogeneous and searching for the certification bodies’ provisions is time-consuming. Thus, creating a common repository collecting certification bodies’ decisions on mHealth apps and the criteria considered can help SR authors.

##### 3.3.3.2. Assessing the integrity of mHealth interventions. Recommendation 12. Use a standard method to measure and summarize intervention integrity of mHealth interventions.

Researchers still need to agree on how to assess mHealth intervention integrity. A framework summarizing intervention integrity per study arm (*e.g.*, high, moderate, or low) would promote assessment consistency, and one needs to be created. As obtaining the overall intervention integrity for the study can be challenging, at least the critical factors should be explicitly reported and judged. Examples of integrity items may be the number of participants receiving the intervention, when and how often the intervention was delivered, whether the intervention was modified during the study, methods used to assess adherence, and the actual observed adherence [25].

##### Recommendation 13. SR authors should plan how to deal with studies with low adherence to the mHealth intervention.

Adherence to an intervention is the degree to which participants use and engage with the intervention as intended. SR authors should carefully plan how to deal with studies with low adherence. For example, if these studies will be eligible or how non-adherent participants will be managed in the analyses and addressed in the risk of bias assessment.

##### Recommendation 14. SR authors should try to extract information on mHealth intervention intensity and use it in the analysis.

Measuring mHealth interventions’ intensity helps to understand their effectiveness and optimize their design. Thus, SR authors should try to extract this information and use it in the analysis, for example, for subgroup analysis or *meta*-regression. However, no consensus exists on the core metrics to summarize mHealth intervention intensity.

#### 3.3.4. Maintaining mHealth SRs up to date

##### Recommendation 15. Processes for updating mHealth SRs should adapt to the dynamic mHealth app market.

The market regularly adds new apps and updates the available ones. Implementing updating processes that reflect this dynamic field is essential to ensure that SRs remain current, applicable, and relevant. Consider developing living systematic reviews, that is, SRs which are “continually updated, incorporating relevant new evidence as it becomes available” [26]. Lessons learned during the COVID-19 pandemic for updating living SRs may be applicable to mHealth living SRs [27].

##### Recommendation 16. Rapid reviews and evidence maps can provide helpful information in mHealth by offering a quicker evidence synthesis and research gap identification.

Rapid reviews and evidence maps are helpful in mHealth, where new technologies are rapidly emerging, and there is a need to keep up with the latest developments. Follow recognised guidance for rapid reviews and evidence map development [28,29].

#### 3.3.5. Recommendations that apply to several review stages

##### Recommendation 17. SRs should try to capture the complexity of mHealth interventions.

Factors contributing to the complexity of mHealth interventions include their integration with other health services, interactions between multiple technology components, and user adherence/engagement. Addressing this complexity in SRs is critical to evaluate mHealth interventions’ effectiveness.

##### Recommendation 18. Consider using a taxonomy of mHealth interventions to improve the clarity, organization, and evaluation of mHealth interventions in SRs.

Using a common taxonomy to classify mHealth interventions would make it easier to apply the review inclusion criteria, determine the intervention and comparator in each study, establish meaningful comparisons, organize the information, evaluate the impact of different interventions, communicate more effectively with others, and identify gaps in the field. An example is the Cochrane Effective Practice and Organisation of Care (EPOC) taxonomy [30], which has been used by Cochrane authors in SRs of health systems interventions.

## 4. Discussion

### 4.1. Summary of main results

Our study identified methodological challenges specific to mHealth SRs and developed consensus-based recommendations to address selected methodological challenges. The aspects most frequently identified as more or much more challenging in our survey were defining the intervention intensity and components, extracting the mHealth intervention details, dealing with dynamic research with continuously evolving interventions, assessing intervention integrity, defining the intervention eligibility, and maintaining the review updated. The workshop focused on mHealth intervention integrity and how to keep mHealth SRs current. Based on the workshop and discussion via email, we developed 18 consensus-based recommendations to address these methodological challenges.

### 4.2. Strengths and limitations

We are confident we identified the main methodological challenges specific to mHealth SRs. First, literature searches informed the survey. Second, only two survey respondents pointed to new challenges not listed in our survey: high heterogeneity among mHealth studies and low quality of mHealth trials. However, we consider these additional challenges not specific to mHealth interventions. Third, the survey respondents had authored at least one mHealth intervention SR and another non-mHealth SR, which helped identify challenges specific to mHealth SRs.

The main survey limitation was its low response rate (5 %), which raises concerns about the representativeness of the challenges and recommendations identified, as they might not fully reflect the experiences of all researchers in the mHealth field. Although contributors to these recommendations are from various regions and cultures, it is important to consider variations in access and use of mobile technology when applying the recommendations. This low response is partially explained by the frequent inoperative emails (13 %). As institutional email addresses can be cancelled very quickly once researchers change institutions, the response rate may increase if SR authors had been contacted via more stable channels, such as ORCID. Other potential explanations for the low response rate are the survey topic specificity and the requirement to have authored at least two SRs (which was not checked before sending the invitations). Moreover, no SR author from Africa or Central/South America participated in the project: we may have missed methodological challenges relevant in these settings.

### 4.3. Comparison with prior work

Our survey is the first one asking SR authors for methodological challenges specific to mHealth interventions. A recent review of mHealth SRs in chronic disease management concluded that the most significant challenges in mHealth intervention development and evaluation were designing high-quality studies, developing robust interventions in combination with health professional input and identifying tools and methods to improve patient adherence [31]. These findings align with ours, but we also identified challenges in keeping SRs current.

### 4.4. Implications for practice and future research

Our study highlights the importance of addressing the methodological challenges related to mHealth intervention integrity in SRs, and calls for collaboration among trialists, systematic reviewers, and guideline developers to achieve this goal. The eighteen recommendations developed in the current study represent a significant asset, but their practical applicability and impact on future mHealth research need to be evaluated.

There is a need to improve the reporting and evaluation of mHealth intervention integrity in RCTs and SRs. This concept is rarely addressed in RCTs, probably due to a lack of agreed-upon definitions and measurements [25]. Moreover, current reporting guidelines omit advice on reporting of mHealth intervention integrity [23,32–36], and how to handle this information is unclear. Still, SRs should try to judge intervention integrity per study arm and integrate this information into the analyses. Developing a framework for quantitatively assessing mHealth interventions’ integrity would facilitate consistent evaluation in SRs. A set of agreed-upon data elements and definitions for extraction of data would be a good starting point towards consistency. The improvement of the evaluation and reporting of mHealth intervention integrity in RCTs and SRs would benefit the broader research community and the quality of synthesized evidence. First, enhancing the transparency and reproducibility of mHealth interventions, by providing clear descriptions of the intervention components, delivery, and adherence. Second, facilitating the comparison of mHealth interventions across different studies and settings, by using standardized definitions and measurements of intervention integrity. Third, assessing the influence of intervention integrity on the outcomes of mHealth interventions, by integrating this information into the analyses of SRs, through techniques such as *meta*-regression. Fourth, identifying the optimal conditions for implementing mHealth interventions, by examining the factors that affect intervention integrity, effectiveness, and sustainability.

## 5. Conclusions

SRs of mHealth interventions present specific methodological challenges compared to non-mHealth interventions, particularly those related to intervention integrity and keeping SRs up to date. Using our recommendations for addressing these challenges can improve evidence identification, assessment, and synthesis in mHealth SRs.

## Supplementary Material

1

2

3

4

5

6

## Figures and Tables

**Table 1:** Characteristics of the survey respondents (n = 50).

Characteristic	n (%)
**Total participants**	50
**Academic background**	
Psychology	14 (28 %)
Medicine	14 (28 %)
Epidemiology/Public Health	13 (26 %)
Computer science	7 (14 %)
Social science	6 (12%)
Sports science	5 (10 %)
Physiotherapy	4 (8 %)
Nursing	3 (6 %)
Engineering	2 (4 %)
Other backgrounds^1^	11 (22%)
**Participants’ experience in mHealth SRs**	
Experienced reviewers (≥ 2 mHealth SRs)	25 (50 %)
Non-experienced reviewers (1 mHealth SR)	25 (50 %)
**Number of mHealth SRs per participant**	
Mean (sd)	2.0 (1.3)
Median (IQR)	2 (1 - 3)
> 3 SRs	8 (16 %)
2-3 SRs	17 (34 %)
1 SRs	25 (50 %)
**Number of non-mHealth SRs per participant**	
Mean (sd)	3.8 (1.5)
Median (IQR)	5 (2.25 - 5)
> 3 mHealth SRs	31 (62 %)
2-3 mHealth SRs	14 (28 %)
1 mHealth SR	5 (10 %)

**IQR:** interquartile range. **sd:** standard deviation. **SR:** systematic review.

1 Other backgrounds (one expert each): biology, chemistry, economics, physics, statistics, nutrition, medical informatics, health science, rehabilitation, infectious diseases, communication science. Participants could have more than one background.

**Table 2:** SR authors perceiving each methodological aspect as more or much more challenging in mHealth SRs.

	Total sample (n=50)	Experienced reviewers^1^ (n=25)	Non-experienced ^2^ (n=25)	Absolute difference^3^
***Formulating the review question***				
1. Defining the eligible study designs	40%	40%	40%	0%
2. Defining the population	12%	16%	8%	8%
3. Defining the intervention	66%	64%	68%	−4%
4. Defining the comparator	63%	60%	65%	−5%
5. Selecting the outcomes	42%	36%	48%	−12%

Finding the evidence: searches and study selection				
6. Availability of appropriate controlled vocabulary (*e.g.*, MesH)	50%	52%	48%	4%
7. Identifying the design of the retrieved studies	32%	32%	32%	0%
8. Dealing with grey literature (*e.g.*, conferences)	44%	41%	48%	−7%

***Data extraction and risk of bias/quality assessment***				
9. Extracting mHealth intervention details	71%	68%	75%	−7%
10. Defining the intervention intensity (dose) and components	85%	80%	91%	−11%
11. Assessing intervention integrity	69%	78%	60%	18%
12. Dealing with co-interventions	60%	65%	55%	10%
13. Assessing the risk of bias of non-randomized intervention studies	42%	45%	38%	7%

***Other challenges***				
14. Writing a review protocol that can anticipate all the scenarios	57%	61%	54%	7%
15. Availability of suitable outcome measurement instruments for trials	60%	64%	57%	7%
16. Verifying the validity of data of the included studies	42%	42%	42%	0%
17- Large amount of missing data	45%	56%	32%	24%
18. Differential follow-up rates between intervention and comparator	37%	35%	39%	−4%
19. Interpreting outcomes and identifying thresholds for decision-making	51%	57%	45%	12%
20. Including real-world evidence data	50%	50%	50%	0%
21. GRADE to assess the certainty of the evidence	44%	53%	37%	16%
22. Dealing with a dynamic research field with continuously evolving interventions	70%	74%	67%	7%
23. Maintaining an updated review	65%	88%	42%	46%
24. Considering preprints (reports without peer-review process)	32%	53%	11%	42%

Additional challenges (free text)	1. Lack of an agreed-upon definition and terminology for mHealth: it is challenging to define what counts as a mHealth intervention.			
	2. Lack of agreed criteria to identify, describe and assess Apps.			

	3. Lack of consensus on how much face-to-face contact is acceptable to define an intervention as mHealth.			
	4. mHealth interventions often include ‘human’ elements as well.			
	5. mHealth interventions are complex, but their theoretical basis needs better reporting. Besides, the actual content of mHealth interventions is often hard to find - in terms of theory/behaviour change techniques and interactivity. Difficult to define what counts as interactive/tailored as well.			
	6. Finding essential information about mHealth interventions is time-consuming: it is disseminated through different reports.			
	7. The optimum study designs to scale mHealth interventions must be established.			
	8. mHealth intervention studies often present large amounts of missing participant data.			
	9. High heterogeneity (*e.g.*, populations, interventions, and control conditions) among mHealth studies makes evidence synthesis difficult.			
	10. Low-quality evidence (model-based studies, very few empirical studies, and poor reporting).			
	11. Lack of agreement on distinguishing efficacy and effectiveness of mHealth trials.			

1 Experienced SR authors: authoring at least two mHealth SRs.

2 Non-experienced SR authors: authoring one mHealth SRs.

3 Absolute differences: Experienced SR authors (%) – Non-experienced SR authors (%).

The colour represents the frequency of each methodological challenge. Green: 0 to 39%. Orange: 40% to 59%. Red: at least 60%. Yellow: absolute difference between experienced and non-experience SR authors of more than 20 percentual points.

**Table 3:** Characteristics of the workshop participants (n = 11).

Characteristic	n (%)
Total participants	11
Female participants	7 (64 %)
Residence geographical area	
Europe	**9 (82 %)**
Switzerland	2 (18 %)
Spain	2 (18 %)
Greece	1 (9 %)
Germany	1 (9 %)
Italy	1 (9 %)
United Kingdom	1 (9 %)
Oceania	**2 (18 %)**
Australia	1 (9 %)
New Zealand	1 (9 %)
Asia	**1 (9 %)**
Singapore	1 (9 %)
Number of mHealth systematic reviews per expert	
> 3	5 (45 %)
2–3	3 (27 %)
1	3 (27 %)
Experts involved in Cochrane	
	2 (18 %)

**Table 4:** Summary table.

**What was already known on the topic** • mHealth is a fast-developing field with an exponential increase in mHealth studies. • Systematic reviews of mHealth interventions may have different methodological challenges than systematic reviews of conventional interventions, such as medications.
**What this study added to our knowledge** • Systematic review authors perceive that systematic reviews of mHealth interventions have specific methodological challenges. • The methodological aspects most frequently identified as challenging were those related to intervention integrity and maintaining the review up to date. • mHealth intervention integrity assessment in systematic reviews requires an agreed definition, reporting and measurement. • Keeping systematic reviews of mHealth interventions current requires updating processes that reflect this dynamic field.

## Box 1 Key mHealth intervention characteristics to report in systematic reviews.

1. mHealth intervention purpose and functions. For example, an intervention for providing reminders to take medication.
2. Target audience. The population for whom the intervention is intended—for example, an intervention targeting individuals with diabetes.
3. Intervention features: material included (audio, visual), behaviour change technique used (if any), etc.
4. App technical specifications: launch date, last version, compatibility with different devices and operating systems, storage and security features.
5. App version considered in the trial.
6. Certification bodies decisions, if obtained.
7. App quality measured with a validated tool, such as the MARS scoring system.
8. Evidence supporting the effectiveness of the app. This information is essential for evaluating the app’s potential impact on health.
9. Cost of the app, including any subscription or in-app purchase fees. This information helps to assess the accessibility of the app, particularly for populations with limited financial resources.
10. App privacy and security features: data collection and storage practices and compliance with data protection regulations.
