# Supplementary material for: Methodological challenges in systematic reviews of mHealth interventions: Survey and consensus-based recommendations
Source: Int J Med Inform. Author manuscript; Available in PMC 2024 Jun 21. (PMC11192046; doi:10.1016/j.ijmedinf.2024.105345)
Supplement: 1 [file NIHMS1994139-supplement-1.docx]

# Appendix 1. Documents consulted to identify methodological challenges potentially relevant in mHealth systematic reviews.

**Search strategies**

Pubmed (1^st^ January 2018 to 1^st^ June 2022): "Telemedicine"[Mesh] OR mHealth[Title] OR eHealth[Title] OR mobileHealth[Title]

EQUATOR (till 1^st^ June 2022): Telemedicine OR mHealth OR eHealth OR mobile Health

**Documents consulted**

We assessed 44 documents for methodological aspects potentially relevant for systematic reviews of mHealth interventions.

[1] Business Insider. The digital health ecosystem. 2019.

[2] European Commission. Green paper on mobile Health ("mHealth"). 2014.

[3] European Commission. Summary report on the public consultation on the green paper on mobile health. 2015.

[4] European Commission. Shaping Europe’s digital future: mHealth. 2020.

[5] IQVIA. Digital Health Trends 2021: innovation, evidence, regulation, and adoption. In: Science IIfHD, editor.2021.

[6] National Institute for Health and Care Excellence (NICE). Evidence standards framework for digital health technologies. 2019.

[7] World Health Organization. mHealth: new horizons for health through mobile technologies: second global survey on eHealth (Global Observatory for eHealth series - Volume 3). Switzerland: WHO Press; 2011.

[8] World Health Organization. EU mHealth Hub Project - Horizon 2020. 2020.

[9] Wolf JA, Moreau JF, Akilov O, Patton T, English JC, 3rd, Ho J, et al. Diagnostic inaccuracy of smartphone applications for melanoma detection. JAMA Dermatol. 2013;149:422-6. https://doi.org/10.1001/jamadermatol.2013.2382

[10] Marcano Belisario JS, Huckvale K, Greenfield G, Car J, Gunn LH. Smartphone and tablet self management apps for asthma. Cochrane Database Syst Rev. 2013:CD010013. https://doi.org/10.1002/14651858.CD010013.pub2

[11] Noura M, Rahmani A, Jahanfar S, Ellis UM. Mobile phone text messaging for the prevention of sexually transmitted infections. Cochrane Database of Systematic Reviews. 2019. https://doi.org/10.1002/14651858.Cd013454

[12] Davies AM MJ. Developing medical apps and mHealth Interventions A guide for researchers, physicians and informaticians Chapter 1: Introduction to mHealth: Springer Cham; 2020. https://doi.org/10.1007/978-3-030-47499-7

[13] Murray E, Hekler EB, Andersson G, Collins LM, Doherty A, Hollis C, et al. Evaluating Digital Health Interventions: Key Questions and Approaches. Am J Prev Med. 2016;51:843-51. https://doi.org/10.1016/j.amepre.2016.06.008

[14] Nahm ES, Bausell B, Resnick B, Covington B, Brennan PF, Mathews R, et al. Online research in older adults: lessons learned from conducting an online randomized controlled trial. Appl Nurs Res. 2011;24:269-75. https://doi.org/10.1016/j.apnr.2009.09.004

[15] Lopez-Alcalde J, Yakoub N, Wolf M, Munder T, von Elm E, Flückiger C, et al. The RIPI-f (Reporting Integrity of Psychological Interventions delivered face-to-face) checklist was developed to guide reporting of treatment integrity in face-to-face psychological interventions. J Clin Epidemiol. 2022;151:65-74. https://doi.org/10.1016/j.jclinepi.2022.07.013

[16] Baker TB, Gustafson DH, Shaw B, Hawkins R, Pingree S, Roberts L, et al. Relevance of CONSORT reporting criteria for research on eHealth interventions. Patient Educ Couns. 2010;81 Suppl:S77-86. https://doi.org/10.1016/j.pec.2010.07.040

[17] Gold SM, Enck P, Hasselmann H, Friede T, Hegerl U, Mohr DC, et al. Control conditions for randomised trials of behavioural interventions in psychiatry: a decision framework. The lancet Psychiatry. 2017;4:725-32. https://doi.org/10.1016/S2215-0366(17)30153-0

[18] Vis C, Bührmann L, Riper H, Ossebaard HC. Health technology assessment frameworks for eHealth: A systematic review. Int J Technol Assess Health Care. 2020;36:204-16. https://doi.org/10.1017/s026646232000015x

[19] Yeaton WH, Sechrest L. Critical dimensions in the choice and maintenance of successful treatments: strength, integrity, and effectiveness. J Consult Clin Psychol. 1981;49:156-67. https://doi.org/10.1037//0022-006x.49.2.156

[20] Perepletchikova F, Treat TA, Kazdin AE. Treatment integrity in psychotherapy research: analysis of the studies and examination of the associated factors. J Consult Clin Psychol. 2007;75:829-41. https://doi.org/10.1037/0022-006x.75.6.829

[21] Perepletchikova F, Hilt LM, Chereji E, Kazdin AE. Barriers to implementing treatment integrity procedures: survey of treatment outcome researchers. J Consult Clin Psychol. 2009;77:212-8. https://doi.org/10.1037/a0015232

[22] Rowland SP, Fitzgerald JE, Holme T, Powell J, McGregor A. What is the clinical value of mHealth for patients? NPJ Digit Med. 2020;3:4. https://doi.org/10.1038/s41746-019-0206-x

[23] Gordon WJ, Landman A, Zhang H, Bates DW. Beyond validation: getting health apps into clinical practice. NPJ Digit Med. 2020;3:14. https://doi.org/10.1038/s41746-019-0212-z

[24] Chib A, Lin SH. Theoretical Advancements in mHealth: A Systematic Review of Mobile Apps. J Health Commun. 2018;23:909-55. https://doi.org/10.1080/10810730.2018.1544676

[25] Varshney U, Singh N, Bourgeois AG, Dube SR. Review, Assess, Classify, and Evaluate (RACE): a framework for studying m-health apps and its application for opioid apps. J Am Med Inform Assoc. 2022;29:520-35. https://doi.org/10.1093/jamia/ocab277

[26] Perepletchikova F. On the Topic of Treatment Integrity. Clin Psychol (New York). 2011;18:148-53. https://doi.org/10.1111/j.1468-2850.2011.01246.x

[27] Agarwal S, LeFevre AE, Lee J, L'Engle K, Mehl G, Sinha C, et al. Guidelines for reporting of health interventions using mobile phones: mobile health (mHealth) evidence reporting and assessment (mERA) checklist. Bmj. 2016;352:i1174. https://doi.org/10.1136/bmj.i1174

[28] Ahmed B, Dannhauser T, Philip N. A systematic review of reviews to identify key research opportunities within the field of eHealth implementation. J Telemed Telecare. 2019;25:276-85. https://doi.org/10.1177/1357633x18768601

[29] Jokinen A, Stolt M, Suhonen R. Ethical issues related to eHealth: An integrative review. Nurs Ethics. 2021;28:253-71. https://doi.org/10.1177/0969733020945765

[30] Rhon DI, Fritz JM, Kerns RD, McGeary DD, Coleman BC, Farrokhi S, et al. TIDieR-telehealth: precision in reporting of telehealth interventions used in clinical trials - unique considerations for the Template for the Intervention Description and Replication (TIDieR) checklist. BMC Med Res Methodol. 2022;22:161. https://doi.org/10.1186/s12874-022-01640-7

[31] Asthana S, Jones R, Sheaff R. Why does the NHS struggle to adopt eHealth innovations? A review of macro, meso and micro factors. BMC Health Serv Res. 2019;19:984. https://doi.org/10.1186/s12913-019-4790-x

[32] Wang Y, Min J, Khuri J, Xue H, Xie B, L AK, et al. Effectiveness of mobile health interventions on diabetes and obesity treatment and management: systematic review of systematic reviews. JMIR Mhealth Uhealth. 2020;8:e15400. https://doi.org/10.2196/15400

[33] Dao KP, De Cocker K, Tong HL, Kocaballi AB, Chow C, Laranjo L. Smartphone-Delivered Ecological Momentary Interventions Based on Ecological Momentary Assessments to Promote Health Behaviors: Systematic Review and Adapted Checklist for Reporting Ecological Momentary Assessment and Intervention Studies. JMIR Mhealth Uhealth. 2021;9:e22890. https://doi.org/10.2196/22890

[34] Aji M, Gordon C, Stratton E, Calvo RA, Bartlett D, Grunstein R, et al. Framework for the Design Engineering and Clinical Implementation and Evaluation of mHealth Apps for Sleep Disturbance: Systematic Review. J Med Internet Res. 2021;23:e24607. https://doi.org/10.2196/24607

[35] Cao W, Milks MW, Liu X, Gregory ME, Addison D, Zhang P, et al. mHealth Interventions for Self-management of Hypertension: Framework and Systematic Review on Engagement, Interactivity, and Tailoring. JMIR Mhealth Uhealth. 2022;10:e29415. https://doi.org/10.2196/29415

[36] Alfawzan N, Christen M, Spitale G, Biller-Andorno N. Privacy, Data Sharing, and Data Security Policies of Women's mHealth Apps: Scoping Review and Content Analysis. JMIR Mhealth Uhealth. 2022;10:e33735. https://doi.org/10.2196/33735

[37] Murray E, Khadjesari Z, White IR, Kalaitzaki E, Godfrey C, McCambridge J, et al. Methodological challenges in online trials. Journal of medical Internet research. 2009;11:e9-e. https://doi.org/10.2196/jmir.1052

[38] Eysenbach G. CONSORT-EHEALTH: improving and standardizing evaluation reports of Web-based and mobile health interventions. J Med Internet Res. 2011;13:e126. https://doi.org/10.2196/jmir.1923

[39] Bender JL, Yue RY, To MJ, Deacken L, Jadad AR. A lot of action, but not in the right direction: systematic review and content analysis of smartphone applications for the prevention, detection, and management of cancer. J Med Internet Res. 2013;15:e287. https://doi.org/10.2196/jmir.2661

[40] Stoyanov SR, Hides L, Kavanagh DJ, Zelenko O, Tjondronegoro D, Mani M. Mobile app rating scale: a new tool for assessing the quality of health mobile apps. JMIR Mhealth Uhealth. 2015;3:e27. https://doi.org/10.2196/mhealth.3422

[41] Masterson Creber RM, Maurer MS, Reading M, Hiraldo G, Hickey KT, Iribarren S. Review and nalysis of existing mobile phone apps to support heart failure symptom monitoring and self-care management using the Mobile Application Rating Scale (MARS). JMIR Mhealth Uhealth. 2016;4:e74. https://doi.org/10.2196/mhealth.5882

[42] Marcolino MS, Oliveira JAQ, D'Agostino M, Ribeiro AL, Alkmim MBM, Novillo-Ortiz D. The impact of mHealth interventions: systematic review of systematic reviews. JMIR Mhealth Uhealth. 2018;6:e23. https://doi.org/10.2196/mhealth.8873

[43] Arthurs N, Tully L, O'Malley G, Browne S. Usability and Engagement Testing of mHealth Apps in Paediatric Obesity: A Narrative Review of Current Literature. Int J Environ Res Public Health. 2022;19. https://doi.org/10.3390/ijerph19031453

[44] Kulkarni P, Kirkham R, McNaney R. Opportunities for Smartphone Sensing in E-Health Research: A Narrative Review. Sensors (Basel). 2022;22. https://doi.org/10.3390/s22103893
