# Supplementary material for: Methodological challenges in systematic reviews of mHealth interventions: Survey and consensus-based recommendations
Source: Int J Med Inform. Author manuscript; Available in PMC 2024 Jun 21. (PMC11192046; doi:10.1016/j.ijmedinf.2024.105345)
Supplement: 2 [file NIHMS1994139-supplement-2.pdf]

CP01

Online survey: Methodological challenges in systematic reviews of mHealth Interventions

Dear researcher

We identified you through a search in Web of Science as author of a systematic review of a mHealth intervention.

This survey aims to identify methodological challenges in systematic reviews evaluating the effects of mHealth interventions. The survey does not cover mHealth in other clinical scenarios, such as health monitoring or diagnosis.

We will use the survey results to inform a consensus workshop. Its aim will be to develop recommendations to address methodological challenges in systematic reviews of mHealth interventions. At the end of this survey, you can indicate whether you are interested in participating in this consensus workshop.

This survey is part of the NIH-funded project: Cochrane Complementary Medicine Field: Resource for Research (2R24AT001293).

The survey is **anonymous** and will take you a **maximum of ten minutes**. You can fill in part of the survey, save it with the button "Pause the interview", and complete it later.

If you have any question, don't hesitate to get in touch with us: survey@usz.ch

Claudia Witt , MD, MBA, **L Susan Wieland**, MPH, PhD & **Jesús López-Alcalde**, MPH

Cochrane Complementary Medicine, University of Maryland School of Medicine, USA & University of Zurich, Switzerland

CP02

Consent to participate in the survey

You are eligible to participate in this survey if you have authored at least two systematic reviews: one should have evaluated the effects of a mHealth intervention and the other one a non-mHealth intervention. We are interested in your perspective on the different methodological challenges in conducting systematic reviews of mHealth and non-mHeath interventions.

Mobile Health, also known as mHealth, is the medical and public health practice supported by mobile devices. Examples of mobile devices are smartphones and tablets.

"I confirm that I meet the requirements to participate in the survey and I agree to take part."

- ◯ Yes
- ◯ No

MC01

Methodological challenges in systematic reviews of mHealth interventions

You can see below a list of methodological aspects that may be challenging in systematic reviews assessing the effects (potential benefits or harms) of mHealth interventions. Please remember that mHealth in other clinical scenarios, such as health monitoring or diagnosis, is not the scope of this survey.

How challenging did you find the methodological aspects in your mHealth systematic review(s) compared to your experience in non-mHealth review(s)?

MC02

Challenges in formulating the review question

How challenging did you find formulating the PICO question in your mHealth systematic reviews?

	much less challenging	less challenging	similar challenges	more challenging	much more challenging	don't know / not applicable
Defining the eligible study designs	○	○	○	○	○	○
Defining the population	○	○	○	○	○	○
Defining the intervention	○	○	○	○	○	○
Defining the comparator	○	○	○	○	○	○
Selecting the outcomes	○	○	○	○	○	○

MC03

Challenges in finding the evidence: searches and study selection

How challenging was finding the evidence in your mHealth systematic reviews?

	much less challenging	less challenging	similar challenges	more challenging	much more challenging	don't know / not applicable
Availability of appropriate controlled vocabulary, such as MesH terms	○	○	○	○	○	○
Identifying the design of the retrieved studies	○	○	○	○	○	○
Dealing with grey literature, such as conferences	○	○	○	○	○	○

MC04

Challenges in data extraction and risk of bias/quality assessment

How challenging did you find data extraction and risk of bias/quality assessment in your mHealth systematic review(s)?

	much less challenging	less challenging	similar challenges	more challenging	much more challenging	don't know / not applicable
Extracting details of the mHealth intervention	○	○	○	○	○	○
Defining the intervention intensity (dose) and components	○	○	○	○	○	○
Assessing intervention integrity (use of the study intervention as intended)	○	○	○	○	○	○
Dealing with co-interventions	○	○	○	○	○	○
Assessing the risk of bias of non-randomized intervention studies, such as pre-post studies	○	○	○	○	○	○

MC05

Other challenges

How challenging did you find the following aspects in your mHealth systematic reviews?

	much less challenging	less challenging	similar challenges	more challenging	much more challenging	don't know / not applicable
Writing a review protocol that can anticipate all the scenarios	○	○	○	○	○	○
Availability of suitable outcome measurement instruments for trials	○	○	○	○	○	○
Verifying the validity of data of the included studies	○	○	○	○	○	○
Large amount of missing data	○	○	○	○	○	○
Differential follow-up rates between the intervention and comparator groups	○	○	○	○	○	○
Interpreting outcomes and identifying thresholds for decision-making	○	○	○	○	○	○
Including real word evidence data	○	○	○	○	○	○
Applying GRADE to assess the certainty of the evidence	○	○	○	○	○	○
Dealing with a dynamic research field in which interventions are continuously evolving	○	○	○	○	○	○
Maintaining an updated review: results are outdated soon after publication	○	○	○	○	○	○
Need to consider preprints, that is, reports that have not completed the peer-review process	○	○	○	○	○	○

MC06

Please, add any other methodological challenges in systematic reviews of mHealth interventions or any other comment you would like to share

---

Page 04

Part2

What is your academic background? MI01

(You can choose multiple answers)

- [ ] Biology
- [ ] Chemistry
- [ ] Computer science
- [ ] Economics
- [ ] Engineering
- [ ] Epidemiology
- [ ] Linguistics
- [ ] Medicine
- [ ] Neuroscience
- [ ] Nursing
- [ ] Philosophy
- [ ] Physics
- [ ] Psychology
- [ ] Physiotherapy
- [ ] Social science
- [ ] Sports science
- [ ] Other
  please specify

How many systematic reviews on mHealth interventions have you authored? MI02

[Please choose]

How many systematic reviews (any topic) have you authored? MI03

[Please choose]

MI04

Cochrane Complementary Medicine will organize a two- or three-hour online workshop to develop recommendations for addressing methodological challenges in systematic reviews of mHealth interventions. The date is yet to be confirmed but the workshop will be held sometime between November 2022 and March 2023.

Would you be interested in participating?

- ◯ Yes (by selecting this option you leave the anonymous part of the survey)
- ◯ No

MI05

Thank you for your interest in our workshop.

Please fill out a contact form, which can be found at **https://befragung.usz.ch/ContactForWorkshop/**

Note: If clicking on the link does not work, please copy the link in a new browser window and enter it in there.

Thank you for completing this survey!

If you have any questions, please contact survey@usz.ch

You can close the browser window or tab now.
