# Supplementary material for: Methodological challenges in systematic reviews of mHealth interventions: Survey and consensus-based recommendations
Source: Int J Med Inform. Author manuscript; Available in PMC 2024 Jun 21. (PMC11192046; doi:10.1016/j.ijmedinf.2024.105345)
Supplement: 3 [file NIHMS1994139-supplement-3.docx]

# Appendix 3. Identification of authors of mHealth systematic reviews

**Aim:** to collect contact emails of authors of mHealth interventions SRs indexed in Web of Science between 1 January 2018 and 17 June 2022

**Step 1. Search in MEDLINE (via PubMed), accessed 17 June 2022**

| #1 | "Mobile Applications"[Mesh] | | | 10,129 |
| --- | --- | --- | --- | --- |
| #2 | (mobile*[ti] OR phone*[ti] OR telephone*[ti] OR smartphone*[ti] OR cellphone*[ti] OR smartwatch*[ti] OR Software[ti] OR Portable[ti]) AND (application*[ti] OR app[ti] OR apps[ti]) | | | 7,384 |
| #3 | ("mobile health"[ti] OR mhealth[ti] OR m-health[ti] OR ehealth[ti] OR e-health[ti]) | | | 7,129 |
| #4 | #1 OR #2 OR #3 | 19,815 |  |  |
| #5 | #4 AND (randomized controlled trial[Publication Type] OR (randomized[Title/Abstract] AND controlled[Title/Abstract] ANDtrial[Title/Abstract])) OR systematic[sb] | | | 3,091 |

**Step 2. Download the results in PMID format**

We opened a Microsoft Word file to modify and replace the line breaks with OR. See list of PMIDs at the end of this file.

**Step 3. Search in WoS (Science Citation Expanded), accessed 17 June 2022**

| #1 | Search records with PMIDs in WoS | 2,818 |
| --- | --- | --- |
| #2 | TI=(“Mobile Application*” OR “Cell Phone*” OR cellphone* OR smarthphone* OR “Handheld computer*”OR smartwatch* OR app OR apps OR “mobile health” OR mhealth OR m-health OR ehealth OR e-health ) OR AK=(“MobileApplication*” OR “Cell Phone*” OR cellphone* OR smarthphone* OR “Handheld computer*” OR smartwatch* OR app OR apps OR “mobile health” OR mhealth OR m-health OR ehealth OR e-health ) OR KP=(“Mobile Application*” OR “CellPhone*” OR cellphone* OR smarthphone* OR “Handheld computer*” OR smartwatch* OR app OR apps OR “mobilehealth” OR mhealth OR m-health OR ehealth OR e-health) | 37,171 |
| #3 | ALL=("clinical trial*" OR random* OR placebo OR trial* OR systematic* OR scoping OR metanalysis OR meta-analysis OR Clinicaltrials.gov OR NCT* OR ICTRP OR “International Clinical Trials Registry Plataform” OR EU-CTR OREU-CTIS OR “EU Clinical Trials registry” OR EudraCT OR “EEA CTA” OR UTN OR “Universal Trial Number” OR PROSPERO) | 3,871,758 |
| #4 | #2 AND #3 | 8,468 |
| #5 | #4 AND (Neurology or Oncology or Nursing or Pharmacology Pharmacy or Nutrition Dietetics or Endocrinology Metabolism or Pediatrics or Psychology or Obstetrics or Gynecology or Rehabilitation or Psychology Clinical or Surgery or Geriatrics Gerontology or Infectious Diseases or Substance Abuse or Peripheral Vascular Disease or Respiratory System or Sport Sciences or Orthopedics or Urology Nephrology or Rheumatology or Engineering Biomedical or Gastroenterology or Hepatology or Psychology Multidisciplinary or Dermatology or Allergy or Dentistry Oral Surgery Medicine or Otorhinolaryngology or Anesthesiology or Gerontology or Primary Health Care or Critical Care Medicine or Emergency Medicine or Audiology Speech Language Pathology or Hematology or Tropical Medicine or Integrative Complementary Medicine or Ophthalmology) (Web of Science categories) | 6,256 |
| #6 | #1 OR #5 | 7475 |

**Step 4. Identification of systematic reviews of mHealth interventions published between 2018 and 2022**

We identified a total of 4,153 eligible SRs.

**Step 5. Identification of authors of systematic reviews of mHealth interventions**

We downloaded the contact details of 953 corresponding authors of these 4,153 SRs.

**Full list of PMIDs**

35706879 OR 35700926 OR 35699991 OR 35698156 OR 35691452 OR 35688586 OR 35685899 OR 35683982 OR 35679193 OR 35679107 OR 35675108 OR 35674176 OR 35672832 OR 35671976 OR 35670987 OR 35669573 OR 35662050 OR 35657298 OR 35643018 OR 35641979 OR 35639672 OR 35638576 OR 35636516 OR 35635752 OR 35635746 OR 35632195 OR 35631202 OR 35622401 OR 35616695 OR 35616514 OR 35612886 OR 35609526 OR 35609313 OR 35607385 OR 35604760 OR 35602145 OR 35596546 OR 35592991 OR 35589362 OR 35582884 OR 35576573 OR 35576569 OR 35576560 OR 35575780 OR 35574579 OR 35572348 OR 35565084 OR 35562656 OR 35560002 OR 35559854 OR 35559648 OR 35550285 OR 35549736 OR 35546412 OR 35544294 OR 35535558 OR 35532971 OR 35525126 OR 35514364 OR 35511229 OR 35510925 OR 35508304 OR 35503654 OR 35503653 OR 35503414 OR 35501981 OR 35501757 OR 35496496 OR 35486437 OR 35485667 OR 35483994 OR 35480536 OR 35476681 OR 35473648 OR 35468093 OR 35468091 OR 35465851 OR 35457775 OR 35457349 OR 35451987 OR 35451977 OR 35451976 OR 35451968 OR 35449509 OR 35438442 OR 35436230 OR 35434267 OR 35422666 OR 35420589 OR 35415743 OR 35413592 OR 35410241 OR 35408166 OR 35395932 OR 35394443 OR 35393874 OR 35392974 OR 35389920 OR 35389873 OR 35389367 OR 35387314 OR 35385395 OR 35384845 OR 35379298 OR 35379294 OR 35378301 OR 35377327 OR 35377325 OR 35357325 OR 35357323 OR 35357311 OR 35355920 OR 35353052 OR 35349576 OR 35349466 OR 35343908 OR 35339412 OR 35335002 OR 35331943 OR 35330427 OR 35329262 OR 35324994 OR 35324452 OR 35319476 OR 35319470 OR 35318189 OR 35317764 OR 35315777 OR 35313935 OR 35311679 OR 35311677 OR 35307306 OR 35297768 OR 35293871 OR 35293867 OR 35293364 OR 35289756 OR 35285809 OR 35284389 OR 35278093 OR 35277436 OR 35271457 OR 35266189 OR 35263798 OR 35262490 OR 35260510 OR 35257698 OR 35255773 OR 35254270 OR 35241140 OR 35238793 OR 35234655 OR 35232456 OR 35230245 OR 35230243 OR 35226699 OR 35221680 OR 35219336 OR 35219309 OR 35212553 OR 35210182 OR 35207359 OR 35206520 OR 35188478 OR 35188475 OR 35188471 OR 35188469 OR 35184262 OR 35182377 OR 35175209 OR 35175202 OR 35173644 OR 35173502 OR 35168981 OR 35168915 OR 35166687 OR 35162351 OR 35156935 OR 35155341 OR 35152244 OR 35148434 OR 35148428 OR 35146942 OR 35144583 OR 35144100 OR 35142630 OR 35141265 OR 35140061 OR 35133634 OR 35130898 OR 35128952 OR 35124473 OR 35122365 OR 35120174 OR 35119373 OR 35119370 OR 35113334 OR 35113034 OR 35113029 OR 35111915 OR 35111332 OR 35107431 OR 35106942 OR 35105640 OR 35103609 OR 35102628 OR 35090520 OR 35090464 OR 35089155 OR 35084356 OR 35084346 OR 35080713 OR 35078238 OR 35077657 OR 35076553 OR 35066706 OR 35065922 OR 35061757 OR 35060913 OR 35060910 OR 35060252 OR 35060222 OR 35057451 OR 35029535 OR 35026491 OR 35025755 OR 35019848 OR 35018280 OR 35014965 OR 35014961 OR 35010884 OR 35010791 OR 35008045 OR 35006076 OR 35005202 OR 34994708 OR 34991593 OR 34989677 OR 34983621 OR 34982724 OR 34982718 OR 34980208 OR 34975710 OR 34971922 OR 34970424 OR 34963562 OR 34963472 OR 34959919 OR 34954099 OR 34953433 OR 34949540 OR 34948546 OR 34941558 OR 34940870 OR 34936374 OR 34932104 OR 34929044 OR 34924318 OR 34915945 OR 34906924 OR 34901428 OR 34896916 OR 34895337 OR 34893387 OR 34890355 OR 34890354 OR 34890353 OR 34890104 OR 34886274 OR 34886039 OR 34878990 OR 34878409 OR 34876067 OR 34875628 OR 34871640 OR 34869915 OR 34869187 OR 34865645 OR 34864830 OR 34864554 OR 34864483 OR 34862449 OR 34861793 OR 34860681 OR 34860673 OR 34860664 OR 34859602 OR 34857035 OR 34856524 OR 34855618 OR 34855609 OR 34854816 OR 34854335 OR 34848798 OR 34847402 OR 34841729 OR 34841554 OR 34840486 OR 34836391 OR 34831725 OR 34825740 OR 34817388 OR 34815242 OR 34812521 OR 34806998 OR 34805387 OR 34802002 OR 34801348 OR 34801006 OR 34800254 OR 34791779 OR 34786870 OR 34786698 OR 34783675 OR 34783674 OR 34779781 OR 34777200 OR 34774015 OR 34770193 OR 34770178 OR 34769570 OR 34768316 OR 34762057 OR 34758761 OR 34753490 OR 34751673 OR 34751662 OR 34747713 OR 34747118 OR 34742357 OR 34738687 OR 34736817 OR 34735603 OR 34735165 OR 34730535 OR 34728250 OR 34726610 OR 34726317 OR 34724874 OR 34723447 OR 34715833 OR 34713162 OR 34710983 OR 34706123 OR 34700074 OR 34694227 OR 34689837 OR 34682962 OR 34674922 OR 34673535 OR 34665845 OR 34661649 OR 34657825 OR 34656190 OR 34647896 OR 34644389 OR 34644350 OR 34643538 OR 34639741 OR 34639692 OR 34639518 OR 34639252 OR 34634096 OR 34631986 OR 34631134 OR 34628540 OR 34624434 OR 34621626 OR 34617916 OR 34617915 OR 34615425 OR 34611287 OR 34610846 OR 34610049 OR 34601832 OR 34599495 OR 34598848 OR 34590978 OR 34585137 OR 34582258 OR 34580067 OR 34574966 OR 34573208 OR 34569327 OR 34567611 OR 34561057 OR 34559058 OR 34559053 OR 34556176 OR 34554814 OR 34550370 OR 34550072 OR 34549333 OR 34547030 OR 34546178 OR 34544569 OR 34544393 OR 34542415 OR 34538272 OR 34537477 OR 34536584 OR 34535608 OR 34535162 OR 34534770 OR 34533466 OR 34530868 OR 34524092 OR 34521400 OR 34519670 OR 34519665 OR 34518568 OR 34508365 OR 34501572 OR 34501492 OR 34499045 OR 34494525 OR 34489366 OR 34487313 OR 34477561 OR 34476415 OR 34474983 OR 34473447 OR 34473068 OR 34472895 OR 34472601 OR 34463631 OR 34463630 OR 34462184 OR 34459905 OR 34453570 OR 34448725 OR 34445995 OR 34444103 OR 34443991 OR 34431478 OR 34429089 OR 34427813 OR 34427188 OR 34425784 OR 34420924 OR 34420922 OR 34420918 OR 34414832 OR 34412727 OR 34410512 OR 34407782 OR 34406971 OR 34405528 OR 34398800 OR 34396685 OR 34392429 OR 34387773 OR 34386838 OR 34385097 OR 34373903 OR 34373299 OR 34371975 OR 34370166 OR 34363130 OR 34356267 OR 34348719 OR 34347865 OR 34345805 OR 34344171 OR 34338710 OR 34336779 OR 34334715 OR 34334288 OR 34334079 OR 34333211 OR 34330073 OR 34328442 OR 34328440 OR 34328423 OR 34324218 OR 34323929 OR 34314254 OR 34313593 OR 34312156 OR 34309576 OR 34309569 OR 34308900 OR 34308256 OR 34299804 OR 34294852 OR 34291892 OR 34287209 OR 34283028 OR 34275463 OR 34274211 OR 34273797 OR 34269681 OR 34269387 OR 34266323 OR 34264696 OR 34259635 OR 34256698 OR 34256131 OR 34255722 OR 34255711 OR 34255698 OR 34246153 OR 34243738 OR 34235246 OR 34234368 OR 34233644 OR 34228735 OR 34224671 OR 34222509 OR 34205744 OR 34197699 OR 34189933 OR 34185013 OR 34184991 OR 34184976 OR 34182157 OR 34180527 OR 34178793 OR 34175270 OR 34174104 OR 34167763 OR 34167566 OR 34161946 OR 34152277 OR 34151710 OR 34151444 OR 34139398 OR 34132227 OR 34128819 OR 34128810 OR 34124856 OR 34124238 OR 34116816 OR 34114965 OR 34114961 OR 34111772 OR 34108892 OR 34106075 OR 34104685 OR 34104627 OR 34104463 OR 34102945 OR 34100234 OR 34098040 OR 34092330 OR 34081234 OR 34081137 OR 34081023 OR 34081019 OR 34076580 OR 34071782 OR 34071342 OR 34065775 OR 34061034 OR 34058610 OR 34057649 OR 34057469 OR 34057427 OR 34052785 OR 34051588 OR 34050095 OR 34047704 OR 34046178 OR 34045994 OR 34039277 OR 34033581 OR 34032583 OR 34021309 OR 34020099 OR 34019624 OR 34018138 OR 34009888 OR 34004469 OR 34003685 OR 34003138 OR 34001705 OR 34001178 OR 33999005 OR 33997280 OR 33991868 OR 33991686 OR 33984372 OR 33980201 OR 33978597 OR 33974019 OR 33971861 OR 33970123 OR 33963477 OR 33962910 OR 33962624 OR 33959378 OR 33957663 OR 33955837 OR 33949243 OR 33946203 OR 33936466 OR 33935152 OR 33931415 OR 33929636 OR 33928755 OR 33928212 OR 33919899 OR 33919758 OR 33918020 OR 33917697 OR 33909607 OR 33908890 OR 33908885 OR 33907721 OR 33905630 OR 33898606 OR 33898595 OR 33890867 OR 33890859 OR 33890855 OR 33888981 OR 33883477 OR 33882601 OR 33882446 OR 33881406 OR 33879089 OR 33878341 OR 33873026 OR 33872436 OR 33872184 OR 33866968 OR 33861204 OR 33853862 OR 33851923 OR 33849611 OR 33847599 OR 33847588 OR 33845880 OR 33845272 OR 33843604 OR 33843593 OR 33839775 OR 33832166 OR 33828846 OR 33826418 OR 33825827 OR 33825795 OR 33824131 OR 33823833 OR 33821958 OR 33818395 OR 33817686 OR 33811021 OR 33809828 OR 33806809 OR 33799055 OR 33797952 OR 33793309 OR 33792555 OR 33792547 OR 33780297 OR 33774008 OR 33771645 OR 33771045 OR 33769123 OR 33768391 OR 33767648 OR 33759801 OR 33758322 OR 33744300 OR 33743385 OR 33740213 OR 33735320 OR 33733257 OR 33729371 OR 33724898 OR 33724494 OR 33724204 OR 33721835 OR 33715537 OR 33709929 OR 33706032 OR 33704075 OR 33693732 OR 33692708 OR 33690144 OR 33688953 OR 33688842 OR 33688711 OR 33685871 OR 33685325 OR 33684712 OR 33674863 OR 33667616 OR 33666561 OR 33666554 OR 33656449 OR 33656448 OR 33655794 OR 33650977 OR 33645416 OR 33635156 OR 33631541 OR 33630873 OR 33625363 OR 33624440 OR 33624337 OR 33622517 OR 33618070 OR 33616547 OR 33616545 OR 33616534 OR 33611239 OR 33605887 OR 33598832 OR 33598308 OR 33595441 OR 33592310 OR 33591290 OR 33591289 OR 33591279 OR 33587045 OR 33587042 OR 33580174 OR 33578819 OR 33578224 OR 33574573 OR 33574002 OR 33573915 OR 33570841 OR 33570801 OR 33568491 OR 33563100 OR 33560244 OR 33559338 OR 33558838 OR 33558625 OR 33557599 OR 33555263 OR 33555259 OR 33547927 OR 33546656 OR 33544370 OR 33539433 OR 33539311 OR 33537637 OR 33534176 OR 33533730 OR 33530342 OR 33528378 OR 33528371 OR 33522978 OR 33519695 OR 33518036 OR 33516964 OR 33516681 OR 33515784 OR 33512318 OR 33512303 OR 33502000 OR 33497833 OR 33492231 OR 33489782 OR 33484628 OR 33483115 OR 33482896 OR 33480852 OR 33478495 OR 33476177 OR 33470941 OR 33468447 OR 33463875 OR 33459598 OR 33453723 OR 33447853 OR 33446036 OR 33444190 OR 33442988 OR 33439142 OR 33439141 OR 33439140 OR 33438494 OR 33437836 OR 33437462 OR 33433024 OR 33430608 OR 33427672 OR 33425667 OR 33423693 OR 33417506 OR 33416510 OR 33416501 OR 33416214 OR 33413304 OR 33411779 OR 33410758 OR 33406130 OR 33401126 OR 33397664 OR 33393925 OR 33393199 OR 33389312 OR 33388336 OR 33386508 OR 33382382 OR 33377565 OR 33376315 OR 33372626 OR 33371027 OR 33368403 OR 33362612 OR 33361244 OR 33359785 OR 33355160 OR 33350732 OR 33347390 OR 33345878 OR 33345659 OR 33343761 OR 33341989 OR 33334577 OR 33325838 OR 33323209 OR 33322678 OR 33320101 OR 33319490 OR 35373021 OR 33315016 OR 33309947 OR 33309486 OR 33306048 OR 33306037 OR 33305826 OR 33298117 OR 33295296 OR 33295292 OR 33294811 OR 33292724 OR 33284129 OR 33280182 OR 33279317 OR 33274726 OR 33271884 OR 33258786 OR 33255982 OR 33249812 OR 33248904 OR 33245288 OR 33242021 OR 33242020 OR 33241162 OR 33238946 OR 33238931 OR 33236109 OR 33234910 OR 33232263 OR 33231555 OR 33228572 OR 33225982 OR 33224743 OR 33217124 OR 33215348 OR 33213398 OR 33212239 OR 33206567 OR 33200413 OR 33198702 OR 33197234 OR 33196634 OR 33196454 OR 33196452 OR 33190189 OR 33190065 OR 33187535 OR 33184151 OR 33180949 OR 33177134 OR 33174849 OR 33171871 OR 33170131 OR 33167300 OR 33161319 OR 33160812 OR 33152010 OR 33151161 OR 33151154 OR 33146620 OR 33142973 OR 33141092 OR 33141091 OR 33136053 OR 33129990 OR 33127288 OR 33124989 OR 33124987 OR 33124984 OR 33123304 OR 33119572 OR 33118956 OR 33118936 OR 33118397 OR 33116352 OR 33115444 OR 33112363 OR 33112249 OR 33112247 OR 33108910 OR 33107832 OR 33103499 OR 33102346 OR 33097097 OR 33097013 OR 33096497 OR 33093013 OR 33085789 OR 33085767 OR 33085510 OR 33084584 OR 33081393 OR 33081070 OR 33079071 OR 33075743 OR 33074161 OR 33073745 OR 33073061 OR 33067856 OR 33067121 OR 33066812 OR 33062041 OR 33055072 OR 33052122 OR 33050974 OR 33048829 OR 33048319 OR 33044542 OR 33044531 OR 33043358 OR 33040590 OR 33040429 OR 33038506 OR 33037989 OR 33036918 OR 33034564 OR 33028335 OR 33023877 OR 33019676 OR 33019274 OR 33012045 OR 33008716 OR 33006569 OR 33006564 OR 32991591 OR 32991303 OR 32990635 OR 32990633 OR 32990629 OR 32988849 OR 32985999 OR 32985439 OR 32985373 OR 32979294 OR 32976515 OR 32972467 OR 32969828 OR 32967717 OR 32967638 OR 32965230 OR 32965227 OR 32962684 OR 32961306 OR 32960998 OR 32955554 OR 32955447 OR 32946257 OR 32945777 OR 32945775 OR 32945774 OR 32940428 OR 32939927 OR 32936087 OR 32936085 OR 32932102 OR 32930640 OR 32921618 OR 32920164 OR 32916983 OR 32916321 OR 32914696 OR 32912305 OR 32909954 OR 32907384 OR 32906632 OR 32902389 OR 32896955 OR 32896632 OR 32890617 OR 32887560 OR 32873114 OR 32870466 OR 32870158 OR 32869511 OR 32865503 OR 32861897 OR 32859100 OR 32858339 OR 32853369 OR 32845553 OR 32844679 OR 32843265 OR 32842830 OR 32825070 OR 32814693 OR 32813666 OR 32811480 OR 32808931 OR 32806583 OR 32804088 OR 32795989 OR 32795144 OR 32794341 OR 32793345 OR 32790051 OR 32788150 OR 32788142 OR 32779223 OR 32779002 OR 32777691 OR 32777664 OR 32772019 OR 32771018 OR 32761174 OR 32755889 OR 32755831 OR 32752026 OR 32746750 OR 32745732 OR 32743950 OR 32742717 OR 32742066 OR 32741789 OR 32741619 OR 32739716 OR 32738662 OR 32738577 OR 32736660 OR 32735225 OR 32735221 OR 32735218 OR 32734929 OR 32734442 OR 32732828 OR 32730310 OR 32729351 OR 32728652 OR 32723718 OR 32720913 OR 32719973 OR 32716299 OR 32708016 OR 32706727 OR 32706724 OR 32706716 OR 32706698 OR 32700147 OR 32699167 OR 32694000 OR 32691659 OR 32690862 OR 32687474 OR 32684106 OR 32683737 OR 32681304 OR 32680441 OR 32677999 OR 32675229 OR 32673267 OR 32673262 OR 32667858 OR 32665344 OR 32664934 OR 32663145 OR 32663140 OR 32663139 OR 32662323 OR 32659107 OR 32656739 OR 32653025 OR 32652863 OR 32637969 OR 32635174 OR 32631080 OR 32630541 OR 32629637 OR 32628688 OR 32628612 OR 32628125 OR 32624013 OR 32623368 OR 32620149 OR 32618115 OR 32613919 OR 32613635 OR 32613525 OR 32613229 OR 32612882 OR 32607820 OR 32605411 OR 32602362 OR 32595546 OR 32589339 OR 32589153 OR 32579120 OR 32578546 OR 32578361 OR 32575482 OR 32572490 OR 32571432 OR 32571123 OR 32570673 OR 32570661 OR 32570615 OR 32563027 OR 32560936 OR 32555981 OR 32549987 OR 32543683 OR 32540842 OR 32536888 OR 32536880 OR 32528665 OR 32525977 OR 32515747 OR 32513646 OR 32513454 OR 32513291 OR 32510462 OR 32509293 OR 32508183 OR 32506922 OR 32501962 OR 32500161 OR 32498995 OR 32493515 OR 32493155 OR 32490849 OR 32490838 OR 32487157 OR 32487065 OR 32480319 OR 32474557 OR 32471469 OR 32470916 OR 32459654 OR 32459653 OR 32459187 OR 32456468 OR 32452817 OR 32452814 OR 32449690 OR 32446158 OR 32444161 OR 32442154 OR 32442132 OR 32441658 OR 32441655 OR 32438338 OR 32437328 OR 32432695 OR 32432550 OR 32427723 OR 32427115 OR 32427114 OR 32421684 OR 32421187 OR 32414368 OR 32412417 OR 32407148 OR 32406989 OR 32406965 OR 32406581 OR 32401221 OR 32399255 OR 32398065 OR 32394772 OR 32390591 OR 32385847 OR 32384423 OR 32383503 OR 32383449 OR 32380972 OR 32379496 OR 32377676 OR 32376347 OR 32375763 OR 32374352 OR 32364506 OR 32362226 OR 32357368 OR 32357125 OR 32352926 OR 32348282 OR 32348270 OR 32348263 OR 32348255 OR 32347805 OR 32343253 OR 32343250 OR 32342662 OR 32339359 OR 32338624 OR 32336996 OR 32336122 OR 32335288 OR 32334955 OR 32329737 OR 32322996 OR 32320936 OR 32320355 OR 32319963 OR 32317000 OR 32314970 OR 32312243 OR 32311957 OR 32310147 OR 32307244 OR 32306847 OR 32301380 OR 32297946 OR 32297588 OR 32292179 OR 32289310 OR 32286685 OR 32283479 OR 32281941 OR 32281936 OR 32281673 OR 32279063 OR 32268161 OR 32265476 OR 32265094 OR 32257648 OR 32255432 OR 32250314 OR 32248935 OR 32247251 OR 32242908 OR 32242760 OR 32241367 OR 32240835 OR 32238810 OR 32238343 OR 32238332 OR 32237937 OR 32234707 OR 32234632 OR 32230769 OR 32229469 OR 32223596 OR 32217503 OR 32217499 OR 32216074 OR 32212412 OR 32208067 OR 32204540 OR 32201165 OR 32196462 OR 32195370 OR 32192020 OR 32187704 OR 32186518 OR 32184315 OR 32184309 OR 32181747 OR 32176191 OR 32175908 OR 32175752 OR 32171937 OR 32167483 OR 32165550 OR 32165549 OR 32164771 OR 32162075 OR 32159519 OR 32154784 OR 32152763 OR 32151291 OR 32150519 OR 32150514 OR 32150141 OR 32149716 OR 32147654 OR 32146218 OR 32143452 OR 32142961 OR 32142514 OR 32141476 OR 32130185 OR 32130184 OR 32130182 OR 32130181 OR 32130178 OR 32130172 OR 32130169 OR 32130167 OR 32130143 OR 32128234 OR 32128233 OR 32124732 OR 32119972 OR 32119093 OR 32114186 OR 32107817 OR 32106713 OR 32101302 OR 32098779 OR 32098739 OR 32095954 OR 32093701 OR 32092160 OR 32087342 OR 32086893 OR 32086525 OR 32085422 OR 32081517 OR 32072838 OR 32069288 OR 32069067 OR 32066490 OR 32061819 OR 32058976 OR 32058140 OR 32055094 OR 32050996 OR 32050044 OR 32049065 OR 32047587 OR 32046499 OR 32041693 OR 32041640 OR 32038153 OR 32035748 OR 32030193 OR 32028926 OR 32026759 OR 32023526 OR 32019529 OR 32014846 OR 32014057 OR 32012674 OR 32012088 OR 32012080 OR 32012038 OR 32012036 OR 32006440 OR 32005778 OR 32005334 OR 32005280 OR 32001517 OR 31991620 OR 31991155 OR 31989852 OR 31985067 OR 31977910 OR 31973787 OR 31973713 OR 31972680 OR 31969272 OR 31965073 OR 31960398 OR 31959326 OR 31959226 OR 31959139 OR 31955942 OR 31955410 OR 31955150 OR 31951215 OR 31948985 OR 31944324 OR 31942918 OR 31942452 OR 31941539 OR 31941475 OR 31941445 OR 31939744 OR 31939515 OR 31938540 OR 31937608 OR 31926063 OR 31924145 OR 31918704 OR 31918340 OR 31915935 OR 31910687 OR 31909896 OR 31904581 OR 31904579 OR 31904578 OR 31890640 OR 31880009 OR 31876765 OR 31871246 OR 31870809 OR 31864348 OR 31856812 OR 31850938 OR 31848158 OR 31848147 OR 31845901 OR 31843639 OR 31842020 OR 31841537 OR 31840286 OR 31838255 OR 31838009 OR 31836333 OR 31831201 OR 31820334 OR 31815678 OR 31815677 OR 31815193 OR 31814140 OR 31813422 OR 31811006 OR 31808376 OR 31807891 OR 31799628 OR 31796480 OR 31796061 OR 31791389 OR 31791295 OR 31790857 OR 31790639 OR 31787410 OR 31785551 OR 31783800 OR 31781525 OR 31778121 OR 31777280 OR 31774402 OR 31773969 OR 31770656 OR 31769758 OR 31769527 OR 31769226 OR 31768261 OR 31762402 OR 31755872 OR 31755869 OR 31753266 OR 31752833 OR 31750837 OR 31747951 OR 31746773 OR 31746766 OR 31743060 OR 31742475 OR 31739848 OR 31738217 OR 31733976 OR 31731292 OR 31730042 OR 31726649 OR 31722151 OR 31721966 OR 31719080 OR 31719030 OR 31718597 OR 31714250 OR 31711969 OR 31711275 OR 31710298 OR 31710269 OR 31706229 OR 31702409 OR 31701021 OR 31699713 OR 31699139 OR 31697244 OR 31694578 OR 31692183 OR 31690536 OR 31689320 OR 31684169 OR 31675540 OR 31671592 OR 31667501 OR 31664959 OR 31662364 OR 31661868 OR 31661080 OR 31658624 OR 31654566 OR 31651407 OR 31651400 OR 31650073 OR 31647821 OR 31647011 OR 31644793 OR 31642088 OR 31638594 OR 31638271 OR 31637367 OR 31634011 OR 31628248 OR 31615456 OR 31607279 OR 31602471 OR 31599729 OR 31593543 OR 31593537 OR 31592979 OR 31584144 OR 31583977 OR 31573939 OR 31573934 OR 31573906 OR 31573903 OR 33323252 OR 31566769 OR 31565245 OR 31561192 OR 31558596 OR 31547909 OR 31542128 OR 31539601 OR 31523905 OR 31523551 OR 31522924 OR 31518324 OR 31518270 OR 31516127 OR 31510755 OR 31509196 OR 31507001 OR 31496664 OR 31492176 OR 31489842 OR 31489841 OR 31486407 OR 31483187 OR 31481605 OR 31477166 OR 31477107 OR 31476994 OR 33323269 OR 31464572 OR 31464192 OR 31464191 OR 31464189 OR 31456578 OR 31454561 OR 31452522 OR 31451942 OR 31448013 OR 31444874 OR 31442241 OR 31439007 OR 31438412 OR 31438349 OR 31437240 OR 31436165 OR 31433259 OR 31431562 OR 31429974 OR 31429644 OR 31425608 OR 31420960 OR 31420956 OR 31416771 OR 31416761 OR 31415448 OR 31414665 OR 31414245 OR 31411147 OR 31407644 OR 31405669 OR 31404051 OR 31404050 OR 31401741 OR 31400105 OR 31399047 OR 31395034 OR 31391133 OR 31389333 OR 31388661 OR 31386151 OR 31384950 OR 31376274 OR 31374033 OR 31373273 OR 31368137 OR 31366398 OR 33323188 OR 31361870 OR 31358537 OR 31353940 OR 31353783 OR 31350681 OR 31342908 OR 31337988 OR 31336101 OR 31335759 OR 31335204 OR 31330023 OR 31326117 OR 31325556 OR 31325288 OR 31322129 OR 31319759 OR 31317869 OR 31317281 OR 31313093 OR 31311573 OR 31309348 OR 31304323 OR 31302077 OR 31299903 OR 31298221 OR 31295907 OR 31293246 OR 31291683 OR 31290404 OR 31277991 OR 31277623 OR 31274112 OR 31269029 OR 31266506 OR 31263555 OR 31260049 OR 31255174 OR 31241192 OR 31240605 OR 31237569 OR 31237240 OR 31235120 OR 31228505 OR 31226579 OR 31222956 OR 31215517 OR 31213866 OR 31208376 OR 31203472 OR 31202003 OR 31199337 OR 31199319 OR 31196062 OR 31192698 OR 31187411 OR 31184922 OR 31182147 OR 31182100 OR 31180750 OR 31180748 OR 31177631 OR 31175963 OR 31174494 OR 31165715 OR 31165709 OR 31162130 OR 31160812 OR 31160014 OR 31159776 OR 31155473 OR 31153917 OR 31153630 OR 31144672 OR 31144666 OR 31144235 OR 31142272 OR 31136332 OR 31132765 OR 31129369 OR 31128958 OR 31128820 OR 31127720 OR 31127719 OR 31125101 OR 31124947 OR 31115348 OR 31112461 OR 31104490 OR 31104034 OR 31102793 OR 31101662 OR 31094354 OR 31093681 OR 31092643 OR 31092044 OR 31088438 OR 31087175 OR 31083767 OR 31082938 OR 31078650 OR 31070388 OR 31067247 OR 31067089 OR 31057081 OR 31042154 OR 31039786 OR 31039580 OR 31038464 OR 31035241 OR 31028038 OR 31019722 OR 31015374 OR 31012860 OR 31008713 OR 31008704 OR 31005588 OR 31003801 OR 31002434 OR 30998227 OR 30998221 OR 30998220 OR 30994846 OR 30994467 OR 30994410 OR 30987685 OR 30985282 OR 30980978 OR 30980006 OR 30979042 OR 30977736 OR 30971374 OR 30969174 OR 30969147 OR 30964435 OR 30961557 OR 30959858 OR 30958833 OR 30953497 OR 30947750 OR 30947636 OR 30946478 OR 30944728 OR 30944143 OR 30943962 OR 30943824 OR 30942695 OR 30938691 OR 30938682 OR 30938681 OR 30931291 OR 30925120 OR 30918040 OR 30916664 OR 30914176 OR 30914003 OR 30913216 OR 30909484 OR 30907929 OR 30905872 OR 30903442 OR 30902428 OR 30901002 OR 30899867 OR 30890120 OR 30888330 OR 30888326 OR 30888321 OR 30883164 OR 30878623 OR 30870770 OR 30869649 OR 30860415 OR 30856542 OR 30852728 OR 30845949 OR 30831331 OR 30829097 OR 30826256 OR 30825094 OR 30824256 OR 30821695 OR 30821692 OR 30819228 OR 30817560 OR 30816851 OR 30815973 OR 30809045 OR 30808389 OR 30804818 OR 30803887 OR 30803885 OR 30799495 OR 30799019 OR 30797722 OR 30796700 OR 30793156 OR 30788692 OR 30788177 OR 30785406 OR 30785404 OR 30783535 OR 30782040 OR 30777847 OR 30776457 OR 30768656 OR 30767906 OR 30761580 OR 30760280 OR 30758294 OR 30758247 OR 30747720 OR 30747715 OR 30745178 OR 30745121 OR 30744697 OR 30744606 OR 30741644 OR 30741641 OR 30740767 OR 30728950 OR 30728064 OR 30726985 OR 30720439 OR 30718125 OR 30715489 OR 30714500 OR 30705956 OR 30702432 OR 30698533 OR 30695078 OR 30694208 OR 30694205 OR 30694200 OR 30688418 OR 30686742 OR 30683710 OR 30681967 OR 30681965 OR 30681964 OR 30679150 OR 30674702 OR 30669986 OR 30664494 OR 30664485 OR 30664480 OR 30664472 OR 30664463 OR 30664457 OR 30659702 OR 30658289 OR 30657334 OR 30654328 OR 30654026 OR 30648217 OR 30640272 OR 30638880 OR 30635865 OR 30635862 OR 30635261 OR 30632972 OR 30632971 OR 30630442 OR 30630108 OR 30629601 OR 30627850 OR 30626196 OR 30624425 OR 30624250 OR 30617044 OR 30617042 OR 30612191 OR 30611977 OR 30609986 OR 30609983 OR 30609978 OR 30609297 OR 30607530 OR 30606197 OR 30602332 OR 30596696 OR 30594232 OR 30593882 OR 30591577 OR 30587857 OR 30586732 OR 30578205 OR 30578185 OR 30577872 OR 30570790 OR 30564435 OR 30559092 OR 30557396 OR 30552249 OR 30552081 OR 30551555 OR 30545806 OR 30545125 OR 30541689 OR 30541536 OR 30522992 OR 30536361 OR 30536149 OR 30535101 OR 30530462 OR 30526560 OR 30526243 OR 30526016 OR 30521685 OR 30507266 OR 30506845 OR 30503602 OR 30514694 OR 30514692 OR 30487115 OR 30486814 OR 30485612 OR 30472970 OR 30470678 OR 30466484 OR 30465630 OR 30458877 OR 30453076 OR 30449261 OR 30447572 OR 30447349 OR 30445989 OR 30445507 OR 30445467 OR 30431384 OR 30431306 OR 30428902 OR 30425028 OR 30414606 OR 30413519 OR 30410742 OR 30408621 OR 30407055 OR 30406378 OR 30404085 OR 30403495 OR 30397934 OR 30392584 OR 30380692 OR 30379128 OR 30375056 OR 30375032 OR 30373727 OR 30368489 OR 30368435 OR 30362363 OR 30361140 OR 30357344 OR 30355551 OR 30349201 OR 30348633 OR 30348142 OR 30347316 OR 30326887 OR 30324496 OR 30316999 OR 30316998 OR 30309837 OR 30309008 OR 30301963 OR 30300870 OR 30294390 OR 30291088 OR 30290276 OR 30289578 OR 30285151 OR 30273901 OR 32021583 OR 30267895 OR 30264583 OR 30262449 OR 30262148 OR 30243751 OR 30239620 OR 30235680 OR 30223139 OR 30213798 OR 30209991 OR 30206519 OR 30204713 OR 30201600 OR 30201060 OR 30190580 OR 30189899 OR 30183463 OR 30181109 OR 30180835 OR 30178072 OR 30175904 OR 30172038 OR 30171827 OR 30161248 OR 30156044 OR 30155796 OR 30155565 OR 30154027 OR 30153253 OR 30152074 OR 30149076 OR 30143479 OR 30143477 OR 30143475 OR 30139724 OR 30139669 OR 30139331 OR 30136644 OR 30135054 OR 30135052 OR 30131313 OR 30130990 OR 30127051 OR 30125790 OR 30121614 OR 30121608 OR 30117784 OR 30111222 OR 30110718 OR 30109154 OR 30109152 OR 30093372 OR 30093368 OR 30092844 OR 30089018 OR 30088110 OR 30086778 OR 30081868 OR 30073445 OR 30072362 OR 30064466 OR 30062632 OR 30061430 OR 30056321 OR 30049464 OR 30047785 OR 30041576 OR 30038502 OR 30037787 OR 30033790 OR 30031553 OR 30026178 OR 30026048 OR 30022583 OR 30021706 OR 30021457 OR 30013857 OR 30012549 OR 30012116 OR 30006326 OR 30006030 OR 29996813 OR 29990679 OR 29990228 OR 29986809 OR 29973252 OR 29973229 OR 29967221 OR 29945861 OR 29944712 OR 29943647 OR 29940921 OR 29939367 OR 29938656 OR 29937195 OR 29937115 OR 29933327 OR 29929949 OR 29927004 OR 29926740 OR 29925497 OR 29925491 OR 29921561 OR 29917096 OR 29914860 OR 29909476 OR 29907244 OR 29903762 OR 29903391 OR 29897921 OR 29895515 OR 29894865 OR 29894774 OR 29871852 OR 29858411 OR 29858041 OR 29857410 OR 29854386 OR 29853435 OR 29851741 OR 29848468 OR 29798832 OR 29793970 OR 29793467 OR 29793397 OR 29788283 OR 29785635 OR 29784635 OR 29784634 OR 29781648 OR 29776899 OR 29775491 OR 29772118 OR 29766596 OR 29763657 OR 29759959 OR 29759957 OR 29756308 OR 29754647 OR 29753356 OR 29748900 OR 29728346 OR 29724054 OR 29723001 OR 29720084 OR 29716883 OR 29710289 OR 29704973 OR 29703242 OR 29703127 OR 29702473 OR 29691983 OR 29691216 OR 29685870 OR 29679398 OR 29677972 OR 29674314 OR 29674307 OR 29674306 OR 29669704 OR 29663265 OR 29662537 OR 29661178 OR 29659413 OR 29656914 OR 29654699 OR 29653532 OR 29643053 OR 29630009 OR 29624123 OR 29622527 OR 29619794 OR 29615421 OR 29607800 OR 29606226 OR 29602430 OR 29601270 OR 29592848 OR 29589050 OR 29582538 OR 29580246 OR 29579015 OR 29578820 OR 29578520 OR 33401367 OR 29566206 OR 29565183 OR 29557547 OR 29555620 OR 29550891 OR 29535081 OR 29533396 OR 29532054 OR 29531280 OR 29530263 OR 29527644 OR 29523502 OR 29523170 OR 29523169 OR 29514344 OR 29513683 OR 29511020 OR 29511007 OR 29510970 OR 29508152 OR 29504207 OR 29500161 OR 29500157 OR 29499663 OR 29494473 OR 29492753 OR 29488798 OR 29483559 OR 29483070 OR 29482636 OR 29482614 OR 29480874 OR 29474702 OR 29470105 OR 29463484 OR 29458358 OR 29455722 OR 29454322 OR 29452259 OR 29449200 OR 29448922 OR 29444335 OR 29439005 OR 29438760 OR 29437546 OR 29433537 OR 29433506 OR 29430455 OR 29425836 OR 29420050 OR 29415699 OR 29415697 OR 29415680 OR 29411270 OR 29406486 OR 29406429 OR 29405923 OR 29402241 OR 29392749 OR 29387266 OR 29386749 OR 29374961 OR 29371177 OR 29370014 OR 29358418 OR 29343463 OR 29335232 OR 29334479 OR 29326406 OR 29326184 OR 29325663 OR 29323626 OR 29322428 OR 29321126 OR 29317380 OR 29316905 OR 29316338 OR 29313363 OR 29310532 OR 29299790 OR 29298749 OR 29295808 OR 29295078 OR 29290888 OR 29289702 OR 29289643 OR 29275969 OR 29267334 OR 29262823 OR 29262693 OR 29258617 OR 29256033 OR 29248628 OR 29247636 OR 29247099 OR 29246532 OR 29243354 OR 29242176 OR 29241520 OR 29241254 OR 29233854 OR 29233806 OR 29233669 OR 29233196 OR 29233169 OR 29232180 OR 29229659 OR 29228934 OR 29227155 OR 29222718 OR 29222595 OR 29212345 OR 29208026 OR 29203459 OR 29198729 OR 29196279 OR 29187412 OR 29187342 OR 29187186 OR 29186803 OR 29184891 OR 29181235 OR 29180345 OR 29175809 OR 29172054 OR 29169546 OR 29155036 OR 29144535 OR 29138050 OR 29129192 OR 29125783 OR 29119719 OR 29116028 OR 29115992 OR 29111887 OR 29103552 OR 29096681 OR 29096614 OR 29090628 OR 29087863 OR 29087256 OR 29077819 OR 29077818 OR 29075430 OR 29074513 OR 29064300 OR 29064141 OR 29061627 OR 29059253 OR 29055449 OR 29051133 OR 29032705 OR 29030327 OR 29030160 OR 29029692 OR 29021128 OR 29020976 OR 29018615 OR 29017191 OR 29016711 OR 28993388 OR 28993302 OR 28992580 OR 28986245 OR 28981460 OR 28973670 OR 28963313 OR 28952379 OR 28950381 OR 28948690 OR 28939582 OR 28936111 OR 28931384 OR 28928110 OR 28923048 OR 28918682 OR 28918669 OR 28918547 OR 28917688 OR 28915825 OR 28914061 OR 28912193 OR 28903892 OR 28894743 OR 28885896 OR 28882743 OR 28880047 OR 28860461 OR 28857576 OR 28855147 OR 28855146 OR 28855063 OR 28851729 OR 28851409 OR 28851342 OR 28847546 OR 28843965 OR 28838887 OR 28836871 OR 28836373 OR 28832243 OR 28830854 OR 28827160 OR 28826348 OR 28821530 OR 28819922 OR 28818820 OR 28810841 OR 28802442 OR 28801393 OR 28800736 OR 28797307 OR 31758759 OR 28795584 OR 28790131 OR 28786320 OR 28768610 OR 28764600 OR 28756791 OR 28752414 OR 28750673 OR 28742913 OR 28736731 OR 28735372 OR 28720103 OR 28718223 OR 28705122 OR 28692830 OR 28687083 OR 28674144 OR 28673323 OR 28666986 OR 28665554 OR 28662834 OR 28659456 OR 28655802 OR 28652227 OR 28645890 OR 28641609 OR 28631933 OR 28630037 OR 28623947 OR 28621202 OR 28619114 OR 28618158 OR 28617171 OR 28612894 OR 28612324 OR 28604138 OR 28600833 OR 28600309 OR 28599674 OR 28597911 OR 28594196 OR 28592069 OR 28583024 OR 28573672 OR 28569933 OR 28567412 OR 28566151 OR 28554914 OR 28550004 OR 28549757 OR 28549455 OR 28546138 OR 28545514 OR 28543566 OR 28541464 OR 28536062 OR 28535331 OR 28532439 OR 28527027 OR 28507010 OR 28506955 OR 28504859 OR 28500020 OR 28494802 OR 28494136 OR 28487932 OR 28487266 OR 28483745 OR 28482322 OR 28481823 OR 28478138 OR 28476273 OR 28471699 OR 28464700 OR 28456513 OR 28454831 OR 28446141 OR 28442453 OR 28441962 OR 28438476 OR 28438144 OR 28428165 OR 28428158 OR 28428157 OR 28423223 OR 28420604 OR 28419149 OR 28400354 OR 28399909 OR 28396303 OR 28389489 OR 28388981 OR 28388923 OR 28385954 OR 28381395 OR 28372580 OR 28370881 OR 28357897 OR 28356720 OR 28350282 OR 28348183 OR 28347663 OR 28338403 OR 28337948 OR 28329223 OR 28328396 OR 28328389 OR 28315480 OR 28302597 OR 28302116 OR 28293621 OR 28292740 OR 28283148 OR 28283147 OR 28281111 OR 28279994 OR 28277820 OR 28276086 OR 28272343 OR 28267047 OR 28264717 OR 28264177 OR 28264176 OR 28258835 OR 28255750 OR 28250552 OR 28249834 OR 28249832 OR 28244548 OR 28237958 OR 28237842 OR 28224445 OR 28223262 OR 28221247 OR 28221061 OR 28220604 OR 28187246 OR 28183341 OR 28174067 OR 28166840 OR 28153814 OR 28152012 OR 28148503 OR 28148473 OR 29871226 OR 28143840 OR 28137701 OR 28132007 OR 28130046 OR 28125970 OR 28106767 OR 28095101 OR 28088674 OR 28085020 OR 28071929 OR 28069560 OR 28055031 OR 28051874 OR 28043176 OR 29645411 OR 28031038 OR 28009342 OR 28003177 OR 27999302 OR 27998876 OR 27993759 OR 27990350 OR 27988093 OR 27987224 OR 27983744 OR 27969026 OR 27956043 OR 27955689 OR 27933450 OR 27927218 OR 27926892 OR 27900995 OR 27900849 OR 27899340 OR 27887610 OR 27886783 OR 29942576 OR 27876082 OR 27869583 OR 27863165 OR 27861583 OR 27856405 OR 27842539 OR 27842473 OR 27837939 OR 27836017 OR 27833070 OR 27830238 OR 27830236 OR 27829576 OR 27826131 OR 27822702 OR 27821601 OR 27818304 OR 27812798 OR 27810633 OR 27664940 OR 27792475 OR 27784506 OR 27783559 OR 27782832 OR 27780796 OR 27778333 OR 27770054 OR 27769282 OR 27766532 OR 27763999 OR 27760883 OR 27760533 OR 27757789 OR 27756288 OR 26631059 OR 27753427 OR 27748662 OR 27742102 OR 27724911 OR 27722974 OR 27721123 OR 27720984 OR 27709734 OR 27707829 OR 27704326 OR 27702408 OR 27702405 OR 27694279 OR 29474713 OR 27659122 OR 27650320 OR 27649587 OR 27634549 OR 27631750 OR 27630020 OR 27613901 OR 27613084 OR 27611958 OR 27611236 OR 27610553 OR 27608677 OR 27601354 OR 27596244 OR 27590255 OR 27587557 OR 27581655 OR 27581172 OR 27573325 OR 27573315 OR 27568126 OR 27554525 OR 27543152 OR 27542825 OR 27542325 OR 27530775 OR 27521811 OR 27520615 OR 27506223 OR 27506148 OR 27502855 OR 27501724 OR 27488411 OR 27486338 OR 27485822 OR 27479493 OR 27473327 OR 27468653 OR 27466046 OR 27464519 OR 27463536 OR 27460771 OR 27454164 OR 27454158 OR 27449226 OR 27448540 OR 27443862 OR 27426247 OR 27425002 OR 27422898 OR 27422639 OR 27421767 OR 27421759 OR 27418275 OR 27417513 OR 27402135 OR 27397753 OR 27392430 OR 27383239 OR 27377792 OR 27377131 OR 27374946 OR 27370229 OR 27354394 OR 27352250 OR 27342791 OR 27335237 OR 27334562 OR 27334418 OR 27334302 OR 27332249 OR 27322089 OR 27321324 OR 27317506 OR 27315764 OR 27301941 OR 27298211 OR 27294708 OR 27287964 OR 27287823 OR 27287668 OR 27260252 OR 27257747 OR 27242162 OR 27239317 OR 27229730 OR 27207721 OR 27192162 OR 27185295 OR 27179726 OR 27174783 OR 27173169 OR 27163250 OR 27157176 OR 27153228 OR 27144393 OR 27140068 OR 27135410 OR 27130166 OR 27128779 OR 27117703 OR 27113598 OR 27098449 OR 27094007 OR 27089351 OR 27073900 OR 27072947 OR 27056905 OR 27054797 OR 27052694 OR 27046668 OR 27043837 OR 27039178 OR 27034992 OR 27018530 OR 27015340 OR 27004719 OR 27001245 OR 27000058 OR 26995281 OR 26988128 OR 26960972 OR 26957103 OR 26956032 OR 26955009 OR 26933140 OR 26932350 OR 26928960 OR 26914371 OR 26907584 OR 26900599 OR 26888421 OR 26888196 OR 26853086 OR 26847379 OR 26822623 OR 26822073 OR 26819969 OR 26813737 OR 26813682 OR 26806712 OR 26799892 OR 26799887 OR 26794197 OR 26787469 OR 26787311 OR 26785637 OR 26781292 OR 26779108 OR 26777408 OR 26772910 OR 26772624 OR 26769910 OR 26769788 OR 26769120 OR 26748792 OR 26744232 OR 26731401 OR 26730560 OR 26729617 OR 26716365 OR 26703120 OR 26701961 OR 26693967 OR 26661717 OR 26653067 OR 26649879 OR 26649177 OR 26628069 OR 26625980 OR 26610389 OR 26589765 OR 26573984 OR 26554314 OR 26553211 OR 26546039 OR 26544175 OR 26540238 OR 26536488 OR 26530929 OR 26513023 OR 26507916 OR 26494256 OR 26494019 OR 26492848 OR 26491263 OR 26490093 OR 26488144 OR 26475670 OR 26452543 OR 26451901 OR 26450209 OR 26400871 OR 26395774 OR 26380263 OR 26379561 OR 26369629 OR 26363048 OR 26362539 OR 26357943 OR 26352854 OR 26333879 OR 26332592 OR 26316499 OR 26308780 OR 26307212 OR 26304621 OR 26303152 OR 26298053 OR 26294453 OR 26290954 OR 26289613 OR 26287926 OR 26283290 OR 26262196 OR 26262016 OR 26251369 OR 26229119 OR 26228390 OR 26220816 OR 26215371 OR 26212714 OR 26209025 OR 26181037 OR 26171733 OR 26159834 OR 26156710 OR 26152999 OR 26151176 OR 26146656 OR 26146515 OR 26125415 OR 26084979 OR 26081864 OR 26078410 OR 26076688 OR 26063161 OR 26048581 OR 26041131 OR 26040644 OR 26040252 OR 26033349 OR 26001926 OR 25997643 OR 25995965 OR 25991158 OR 25991154 OR 25986294 OR 25980866 OR 25963607 OR 25963412 OR 25959301 OR 25947578 OR 25928161 OR 25924825 OR 25917824 OR 25916831 OR 25887998 OR 25884824 OR 25883164 OR 25881735 OR 25872754 OR 25856270 OR 25845672 OR 25841251 OR 25831265 OR 25803705 OR 25803266 OR 25800698 OR 25799001 OR 25790903 OR 25753009 OR 25751133 OR 25740350 OR 25732936 OR 25727678 OR 25720675 OR 25710808 OR 25708251 OR 25680388 OR 25673013 OR 25666218 OR 25649783 OR 25648301 OR 25630361 OR 25629819 OR 25629710 OR 25625810 OR 25624309 OR 25600193 OR 25584730 OR 25582097 OR 25567831 OR 25565587 OR 25565416 OR 25563359 OR 25555099 OR 26958294 OR 25540089 OR 25539976 OR 25513813 OR 25511185 OR 25504143 OR 25502745 OR 25500281 OR 25499872 OR 25499592 OR 25480528 OR 25477073 OR 25474190 OR 25473176 OR 25472682 OR 25469795 OR 25431855 OR 25409001 OR 25402403 OR 25402398 OR 25397860 OR 25396767 OR 25386719 OR 25376414 OR 25355131 OR 25335439 OR 25326074 OR 25310749 OR 25295771 OR 25290556 OR 25283352 OR 25264974 OR 25257518 OR 25217039 OR 25211571 OR 25209729 OR 25196411 OR 25175787 OR 25161104 OR 25138438 OR 25134692 OR 25121620 OR 25103183 OR 25098337 OR 25085225 OR 25075243 OR 25071063 OR 25042159 OR 25037644 OR 25004915 OR 25004456 OR 25000742 OR 25000269 OR 24999983 OR 24985342 OR 24982490 OR 24958355 OR 24943550 OR 24927745 OR 24916567 OR 24904909 OR 24881829 OR 24862459 OR 24846621 OR 24844445 OR 24828126 OR 24807502 OR 24780934 OR 24742381 OR 24739471 OR 24718852 OR 24704648 OR 24671165 OR 24668048 OR 24654927 OR 24652032 OR 24650843 OR 24645829 OR 24642082 OR 24628961 OR 24610324 OR 24604571 OR 24578074 OR 24556530 OR 24519346 OR 24511914 OR 24494974 OR 24486732 OR 24485327 OR 24434827 OR 24427456 OR 24413342 OR 24396050 OR 24393146 OR 25954342 OR 24366061 OR 24363921 OR 24351088 OR 24350804 OR 24330604 OR 24294327 OR 24293120 OR 24282112 OR 24246618 OR 24245838 OR 24245493 OR 24225332 OR 24225149 OR 24215774 OR 24205809 OR 24199747 OR 24177488 OR 24133558 OR 24131553 OR 24095951 OR 24082784 OR 24050486 OR 24050427 OR 24007452 OR 24035143 OR 24015984 OR 24004517 OR 23992038 OR 23952787 OR 23920939 OR 23915067 OR 23911170 OR 23867222 OR 23803275 OR 23782796 OR 23776544 OR 23750773 OR 23697600 OR 23683104 OR 23637534 OR 23608679 OR 23598293 OR 23587561 OR 23563184 OR 23520213 OR 23512821 OR 23507561 OR 23458994 OR 23439183 OR 23429637 OR 23410658 OR 23394465 OR 23355273 OR 23349621 OR 23311452 OR 23186301 OR 23181985 OR 23181455 OR 23285508 OR 23038130 OR 23022989 OR 22958799 OR 22835804 OR 22826375 OR 22802269 OR 22781312 OR 22704741 OR 22646848 OR 22607302 OR 22589569 OR 22548603 OR 22548595 OR 22548594 OR 22521719 OR 22520519 OR 22438297 OR 22268960 OR 22186428 OR 22155629 OR 22138112 OR 22123031 OR 22059446 OR 22052161 OR 21843846 OR 21718092 OR 21707807 OR 21654869 OR 21401537 OR 21267058 OR 21169619 OR 21071584 OR 20429947 OR 20348068 OR 20302678 OR 19965037 OR 19515862 OR 19127002 OR 18605651 OR 18562447 OR 17921245 OR 17466815 OR 17452068 OR 17265704 OR 16884565 OR 16375790 OR 15881824 OR 15829471 OR 15813712 OR 15233773 OR 12622345 (PubMed ID)
