# Supplementary material for: Methodological challenges in systematic reviews of mHealth interventions: Survey and consensus-based recommendations
Source: Int J Med Inform. Author manuscript; Available in PMC 2024 Jun 21. (PMC11192046; doi:10.1016/j.ijmedinf.2024.105345)

Cochrane Complementary Medicine

Trusted evidence.
Informed decisions.
Better health.

Online workshop: Methodological challenges in systematic reviews of mHealth interventions

25 January 2023

13:00 – 15:00 h CET

L. Susan Wieland
Claudia M. Witt
Jesús López-Alcalde

Cochrane Complementary Medicine
University of Maryland School of Medicine
University of Zurich and University Hospital Zurich

USZ Universitäts Spital Zürich

Universität Zürich UZH

University of Maryland School of Medicine

Recording of the workshop

The workshop will be recorded for summarising the results.
The recording will be only used internal purposes.

If anyone objects, please speak up.

Agenda

25 Jan 2023. 13:00 – 15:00 h CET

Cochrane Complementary Medicine

1	2	3	4
**Introduction of participants**	**Project presentation**	**Developing guidance**	**Closing remarks**
13:00 – 13:15	13:15 – 13:30	13:30 – 14:50	14:50 – 15:00

1. Introduction of participants

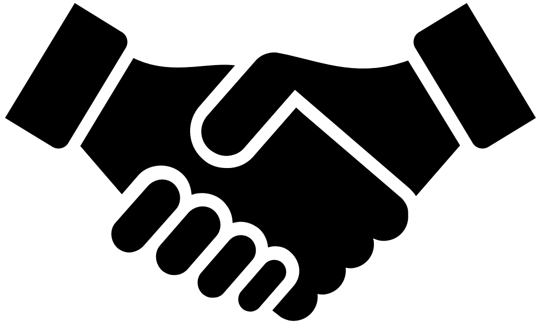

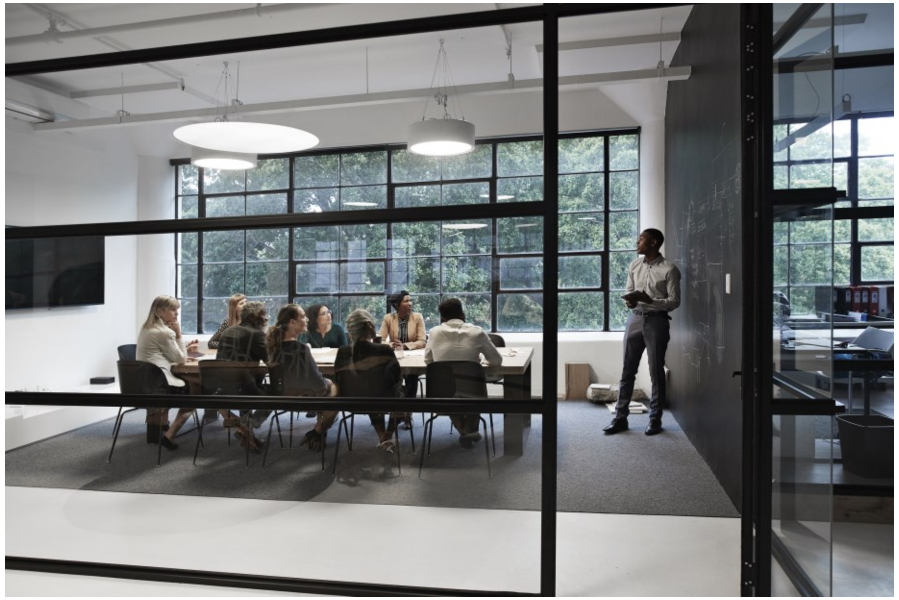

- Name
- Country and institution

Agenda

25 Jan 2023. 13:00 – 15:00 h CET

Cochrane Complementary Medicine

1. **Introduction of participants** — 13:00 – 13:15
2. **Project presentation** — 13:15 – 13:30
3. **Developing guidance** — 13:30 – 14:50
4. **Closing remarks** — 14:50 – 15:00

USZ Universitäts Spital Zürich

Universität Zürich[UZH]

University of Maryland School of Medicine

a. Scope of the survey

01 Clinical question — **Effects of health interventions**
- Other clinical questions excluded
- Examples: health monitoring, dx or prognosis

02 Intervention — **Mobile Health (mHealth) interventions**
- Health interventions supported by mobile devices

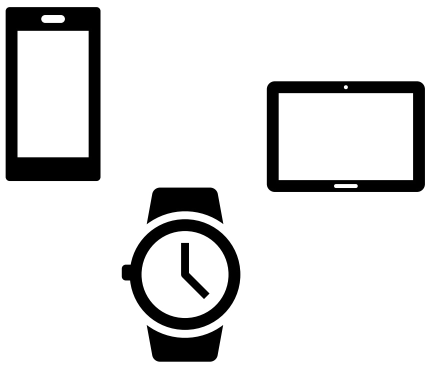

03 Design — **Systematic review**

04 Survey aim — To **identify methodological challenges** specific to SRs evaluating the effects of mHealth interventions

b. Timeline: work done and following steps

Sept – Nov 2022	2023
To **identify** methodological challenges in SRs of mHealth interventions	To **develop recommendations** to overcome the most relevant challenges identified

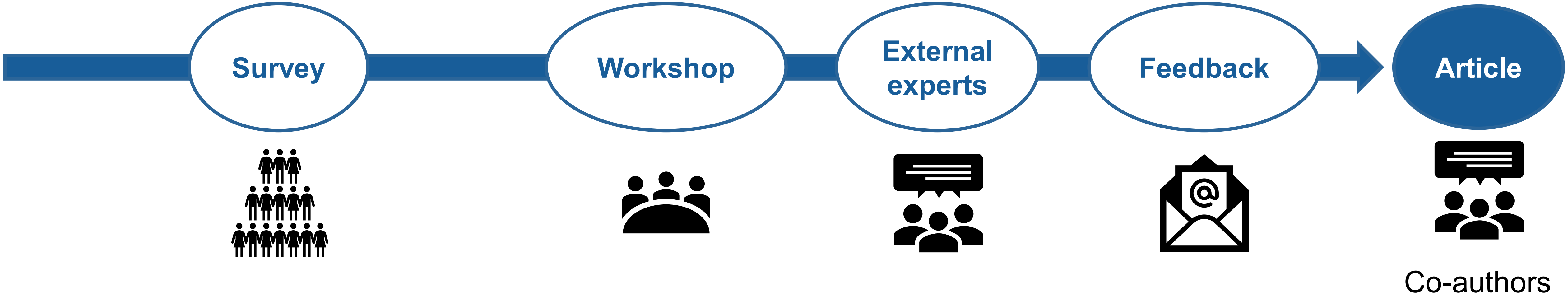

c. Methodological challenges in mHealth interventions SRs: survey preliminary results

01 Survey sample size:

- Total participants: 51
- Experienced systematic reviewers (authoring ≥2 SRs): 25

02 Most challenging areas in SRs of mHealth interventions:

**Keeping the systematic review up to date**	Incorporate relevant new evidence as it becomes available
**Aspects related to the mHealth intervention itself**	Intervention integrity

Criteria for workshop topic selection:

1. Answers of experienced reviewers
2. Topics uncovered in relevant methodological guidance*:
   - mERA reporting checklist
   - CREMAIs reporting guideline
   - CONSORT-EHEALTH
   - TIDieR-telehealth
   - MARS (Mobile app rating scale)

* Based on a non-systematic review

Concept: intervention integrity

Proposed definition

The degree to which the study intervention was delivered as intended[1-5]

Components of intervention integrity

Intervention delivery ⟺ Participant receiving the intervention

Intervention delivery:
- Device
- App
- Internet access
- Health provider

Participant receiving the intervention:
- Adherence
- Withdrawals

[1] Perepletchikova F. Clin Psychol (New York). 2011;18(2):148-153.
[2] Perepletchikova F, et al. J Consult Clin Psychol. 2009;77(2):212-218.
[3] Perepletchikova F, et al. J Consult Clin Psychol. 2007;75(6):829-841.
[4] Yeaton WH, et al. J Consult Clin Psychol. 1981;49(2):156-167.
[5] Lopez-Alcalde J, et al. J Clin Epidemiol. 2022;151:65-74.

Experienced reviewers considering each aspect "More challenging" or "Much more challenging" in mHealth reviews

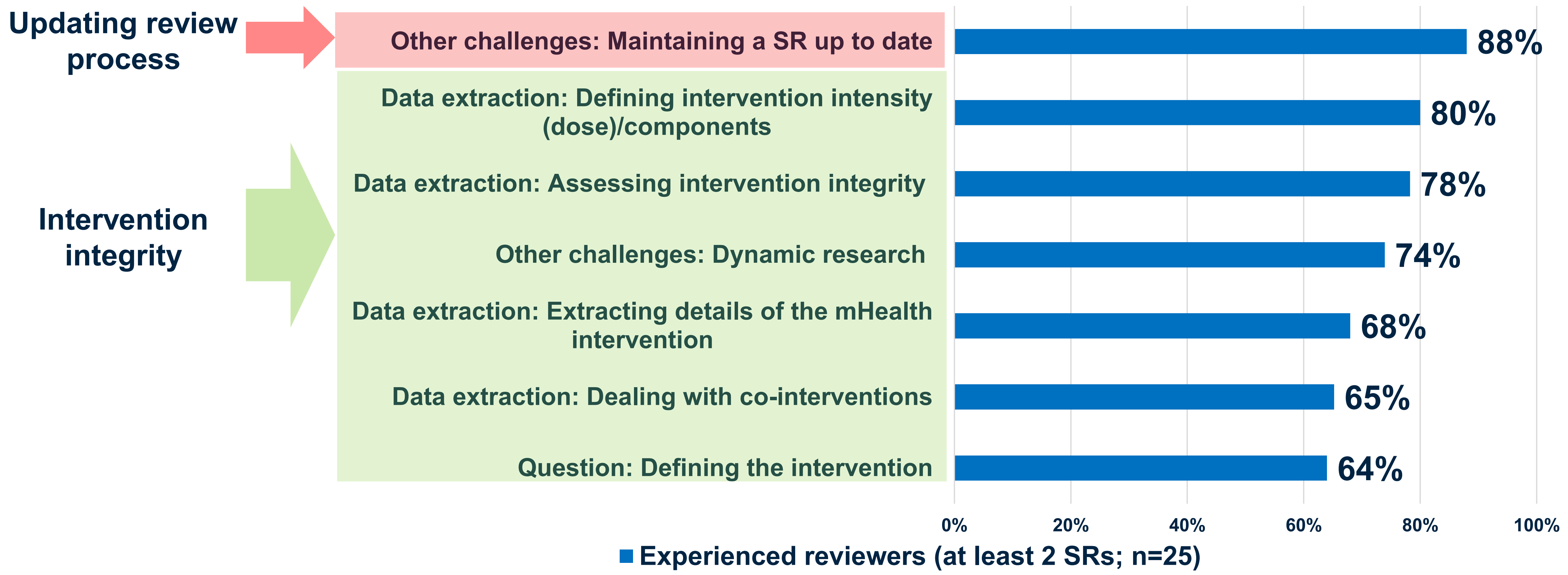

d. Workshop aim

To develop guidance to overcome methodological challenges related to two aspects:

1. Integrity of mHealth interventions	13:30 – 14:10
**Break**	14:10 – 14:20
2. Maintaing mHealth systematic reviews up to date	14:20 – 14:50

Agenda

25 Jan 2023. 13:00 – 15:00 h CET

Cochrane Complementary Medicine

1. **Introduction of participants** — 13:00 – 13:15
2. **Project presentation** — 13:15 – 13:30
3. **Developing guidance** — 13:30 – 14:50
4. **Closing remarks** — 14:50 – 15:00

USZ Universitäts Spital Zürich

Universität Zürich[UZH]

University of Maryland School of Medicine

Topic: Intervention integrity

How did you deal with the following challenges?

Category	#	Challenge	%
**Question formulation**	1	Defining the intervention	64%
**Data extraction of each included study**	2	Extracting details of the mHealth intervention	68%
	3	Defining intervention intensity (dose) / components	80%
	4	Assessing intervention integrity	78%
	5	Dealing with co-interventions	65%
**Other challenges**	6	Dealing with a dynamic field	74%

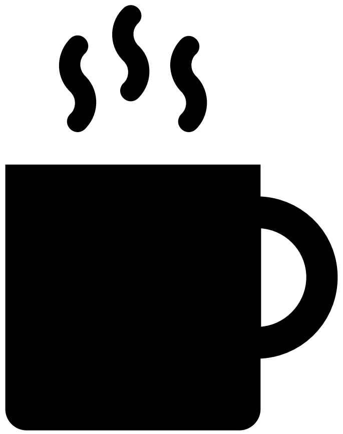

Topic: Maintaining the systematic review up to date

Living systematic review: systematic review which is continually updated, incorporating relevant new evidence as it becomes available (Cochrane definition).

Solutions for maintaining SRs of mHealth interventions up to date? Examples

1. **How to deal with obsolescence of mHealth interventions?**
2. **What sources should be searched?**
3. **Are artificial intelligence approaches useful?**
4. **Others**

5. Closing remarks

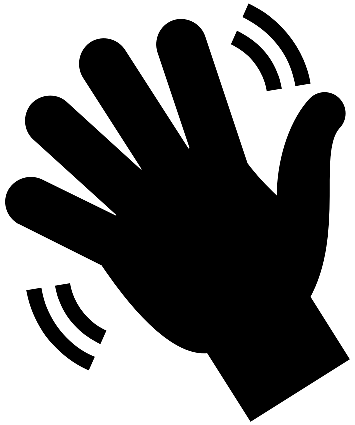

Thank you for your support!

For more information:
Jesús López-Alcalde
Jesus.lopez@usz.ch

USZ Universitäts Spital Zürich

Universität Zürich[UZH]

UNIVERSITY of MARYLAND SCHOOL OF MEDICINE

Cochrane Complementary Medicine

Full reference list

1. Agarwal S, et al. Guidelines for reporting of health interventions using mobile phones: mobile health (mHealth) evidence reporting and assessment (mERA) checklist. Bmj. 2016;352:i1174.

2. Dao KP, et al. Smartphone-Delivered Ecological Momentary Interventions Based on Ecological Momentary Assessments to Promote Health Behaviors: Systematic Review and Adapted Checklist for Reporting Ecological Momentary Assessment and Intervention Studies. JMIR Mhealth Uhealth. 2021;9(11):e22890.

3. Eysenbach G. CONSORT-EHEALTH: improving and standardizing evaluation reports of Web-based and mobile health interventions. J Med Internet Res. 2011;13(4):e126.

4. Rhon DI, et al. TIDieR-telehealth: precision in reporting of telehealth interventions used in clinical trials - unique considerations for the Template for the Intervention Description and Replication (TIDieR) checklist. BMC Med Res Methodol. 2022;22(1):161.

5. Stoyanov SR, et al. Mobile app rating scale: a new tool for assessing the quality of health mobile apps. JMIR Mhealth Uhealth. 2015;3(1):e27.

Methodological challenges in SRs of mHealth interventions: preliminary results in experienced reviewers

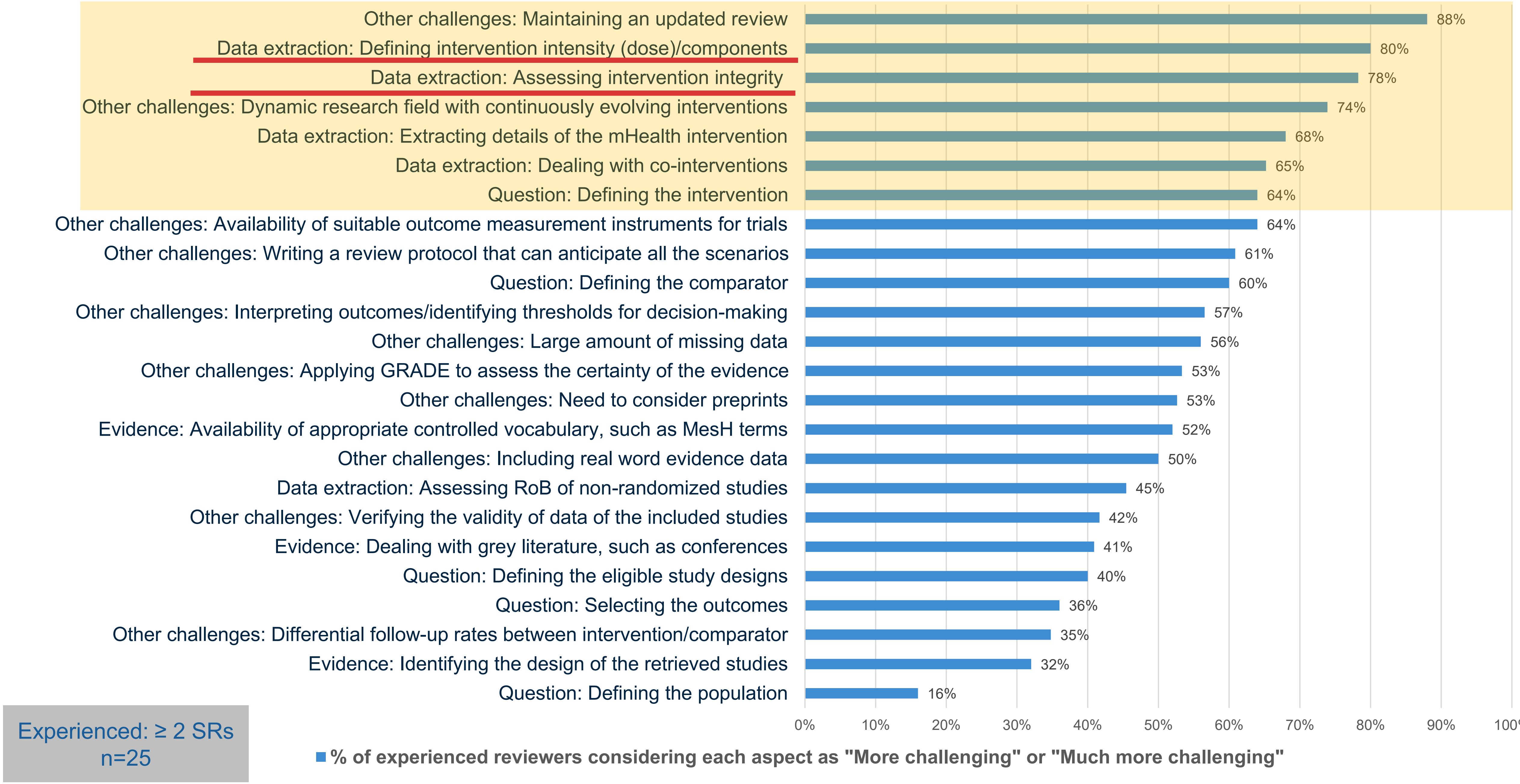

Supplement: 4 [file NIHMS1994139-supplement-4.pdf]
