# Supplementary material for: Methodological challenges in systematic reviews of mHealth interventions: Survey and consensus-based recommendations
Source: Int J Med Inform. Author manuscript; Available in PMC 2024 Jun 21. (PMC11192046; doi:10.1016/j.ijmedinf.2024.105345)
Supplement: 6 [file NIHMS1994139-supplement-6.docx]

**Appendix 6.** **Recommendations for addressing methodological challenges specific to mHealth SRs**

**I. Definition of the eligible mHealth intervention**

*Recommendation 1. Consider performing/consulting a scoping review to inform the protocol of the mHealth intervention SR.*

A scoping review is an evidence synthesis that provides an overview of research evidence and gaps across various fields. Scoping reviews can clarify critical concepts/definitions in the literature and identify key characteristics or factors related to a concept."^1,p950^. They can provide valuable information to define the research question in the review protocol, such as by informing the eligible mHealth interventions and outcomes, and ensuring the review is comprehensive, up-to-date, and relevant. We recommend following recent scoping review guidance, such as the Joanna Briggs Institute (JBI) Manual for Evidence Synthesis [1], in order to assess the appropriateness of the scoping review, the extraction, analysis, and presentation of the results, as well as the implications for clinical practice and research.

*Recommendation 2. The SR eligibility criteria should clearly describe the eligible mHealth intervention, including details of the app.*

SR authors should define inclusion criteria clearly and predetermine how to deal with poor reporting in primary studies. As part of the inclusion criteria, authors should specify the minimum characteristics an app should have to be eligible. There is a need to standardize and develop a comprehensive mHealth app assessment tool beyond MARS and ABACUS to assist in this [2-4]. The protocol should also state whether studies of no longer functioning apps or operating systems will be eligible, and if they will be combined with studies of working apps or examined separately. This specification is important for two reasons: 1) Relevance of the review findings: prioritizing working apps ensures that the review results are relevant to current practice and have more impact. On the other hand, this decision excludes previous evidence that may provide helpful indirect evidence; 2) Workload: the increasing available evidence, including outdated technology, can compromise the workforce of the review team. Thus, excluding studies with no working app versions will help deliver mHealth SRs faster.

*Recommendation 3. mHealth intervention SRs should define the technical context in which the review findings will be applied.*

When defining the eligibility criteria, considering the technical context can help ensure the review findings are relevant and feasible for the intended audience. For example, the context can inform what types of mHealth interventions are most relevant and appropriate: mHealth interventions requiring high-speed internet may not be feasible in remote areas with limited infrastructure.

*Recommendation 4. Do not neglect low-cost and low-tech Health interventions by default.*

Including low-cost and low-tech mHealth interventions in SRs can help to ensure that the solutions are accessible, feasible, cost-effective, and sustainable for different health care settings, regardless of their level of technology or infrastructure. For example, a text message-based intervention may be more feasible than a mobile app, as it requires fewer resources and is easier to scale up.

**II. Search methods**

*Recommendation 5. Develop validated filters for search strategies of mHealth interventions and use of automation screening tools.*

Search filters are used to identify studies based on specific characteristics. A filter for mHealth intervention studies would be helpful because they improve the efficiency of the search process by reducing the number of irrelevant studies that need to be screened. Search strategies using a filter for identifying mHealth intervention studies would improve the efficiency of the search process by reducing the number of irrelevant studies to screen[5]. Search filters to find articles on mHealth interventions should be developed and validated according to rigorous methods, such as appraisal checklists [6]. Consider also using SR software with automation tools for increasing screening efficiency, such as EPPI-Reviewer, DistillerSR, Covidence or Rayyan.

*Recommendation 6. Carefully select the sources to search in mHealth SRs*

Specific search methods guidance will help to overcome mHealth SRs challenges: 1) Diverse dissemination channels in addition to academic journals (*e.g.*, conference proceedings, grey literature, and online databases) and heterogeneous disciplines (*e.g.*, medicine, public health, psychology, engineering, computer science, and health informatics); 2) Unstandardized and heterogeneous terminology, which can lead to inconsistencies in search results; 3) Rapidly evolving field, which requires periodic searches to maintain the review up-to-date; 4) Quality control: searches should prioritize to sources collecting studies with a minimum of quality; and 5) Complexity: the mHealth field presents different technologies, interventions, and outcomes. Identifying the evidence for each of these aspects can be challenging. In this line, it is critical to involve a specialized librarian to choose the most relevant sources to be searched, both bibliographic databases or other sources, such as searching for grey literature.

**II. Data extraction**

**II.1 Extracting the mHealth intervention details**

*Recommendation 7. SR authors should follow standardized data extraction and description of mHealth apps. Available reporting guidelines can help.*

Standardized templates can guide the data extraction of mHealth interventions. They help ensure that the data collected is accurate, complete, consistent, and transparent, making it easier to compare and synthesize findings across studies. Besides, the availability of pre-designed data extraction forms can accelerate SR processes. Reviewers should identify tools to guide the data extraction. Examples are TIDieR-telehealth[7], CONSORT eHealth [8], and the Mobile Application Rating Scale (MARS)[9].

The minimal list of characteristics that reviewers should extract from each mHealth intervention is not agreed upon. Moreover, in MHealth interventions' data extraction can tackle many domains. For example, the app quality, functionality and content, the behavioural change technique implemented, etc. Box 2 proposes features to provide a comprehensive picture of the mHealth intervention and its potential impact on health.

*Recommendation 8. The review team should schedule time and training to extract the mHealth intervention characteristics.*

Setting aside adequate time to extract information about mHealth interventions is essential due to the complexity of mHealth intervention data and the diverse information sources. Two reviewers should typically perform data extraction to increase consistency and minimize errors. The review team should set aside adequate time to train the reviewers, measure the interrater reliability of their judgments, and reach consensus. Consider using tools for estimating how long the review will take to complete, such as PredicTER (Predicting Time requirements for Evidence Reviews) [10].

*Recommendation 9. Make data extraction forms publicly available*

SR authors should make their data extraction forms publicly available and be explicit about any modifications made to established forms. This will save time for other SR authors since developing and piloting data extraction forms in mHealth SRs can be time-consuming. Consider making data extraction forms available in open repositories such as Figshare, Open Science Framework or Zenodo.

*Recommendation 10. Data extraction of mHealth interventions should consider at least two levels: the intervention itself and the app specifications.*

The review team should specify if the review will assess an app or the effects of the mHealth intervention. Thus, collecting information about the mHealth intervention should consider at least two levels: 1) The description of the complex intervention, that is, the target population, intervention goals and components; 2) The description of the app itself, which includes the app's features and version, functionality, availability, and country of use, as these factors can also impact the intervention's effectiveness and implementation.

Collecting information about the target population, goals, and components of the intervention, as well as the app's features, functionality, and usability, is critical to understanding the intervention's effectiveness and potential for implementation in different settings. This information can help identify effective intervention components, inform future efforts to improve the intervention or develop new ones, and identify potential implementation barriers or facilitators. It is also essential to report the app version, availability, and country of use, as these factors can impact the intervention's effectiveness.

*Recommendation 11. Repositories collecting certification bodies' decisions on mHealth apps can help SR authors.*

Certification bodies can have helpful information for authors of mHealth SRs. However, the certification criteria are heterogeneous and searching for the certification bodies' provisions is time-consuming. Thus, creating a common repository collecting certification bodies' decisions on mHealth apps and the criteria considered can help SR authors.

**III.2. Assessing the integrity of mHealth interventions**

*Recommendation 12. Use a standard method to measure and summarize intervention integrity of mHealth interventions.*

Intervention integrity refers to the degree to which the intervention was implemented as intended. Assessing the integrity of the mHealth intervention evaluated in a RCT is essential for evaluating the study's risk of bias and the intervention's applicability and acceptability. However, researchers still need to agree on how to assess mHealth intervention integrity. Assessment should cover common domains such as session attendance and protocol adherence, as well as specific aspects related to technical integrity, including reliable software and hardware, interoperability, user experience, and technology performance and scalability.

A framework to summarise mHealth intervention integrity per study arm (for example, as high, moderate, or low) would promote assessment consistency. As obtaining the overall intervention integrity per study and, later, for the assessed body of evidence, can be challenging, at least the critical factors considered in the assessment should be explicitly judged and reported. Examples of critical items concerning integrity may be the number of participants receiving the intervention, how and where the intervention was delivered, when and how often the intervention was delivered, whether the intervention was tailored or modified during the study, methods to assess adherence, and the actual adherence [11].

*Recommendation 13. SR authors should plan how to deal with studies with low adherence to the mHealth intervention.*

Adherence to an intervention refers to the degree to which participants use and engage with the intervention as intended. Low adherence is common in mHealth studies [12] and can limit the study's statistical power, internal validity, and intervention effectiveness, as well as its generalizability, scalability, and sustainability. Reviewers should carefully plan how to deal with low adherence rates during the review process. For example, they should define if studies with very low adherence rates will be eligible, plan analysis methods for non-adherent participants, and report adherence rates in the included studies.

*Recommendation 14. SR authors should try to extract information on mHealth intervention intensity and use it in the analysis.*

Knowing the intensity of mHealth interventions helps understand their effectiveness and optimize their design. However, no consensus exists on the core metrics to reflect and summarize mHealth intervention intensity. In addition, mHealth RCTs do not follow the usual phases of drug development trials in which the adequate dose of the drug is assessed before efficacy trials. Several metrics can be used to measure mHealth interventions' intensity, including usage, engagement, content, reach, and outcome metrics. These metrics can reflect the frequency of use, the degree of user interaction, the quality and relevance of the information provided, the extent of dissemination in the target population, and the intervention's impact on health outcomes. Thus, SR authors should try to extract this information and use it in the analysis, for example, for subgroup analysis or meta-regression. However, no consensus exists on the core metrics to summarize mHealth intervention intensity.

**IV. Maintaining SRs of mHealth interventions up to date**

*Recommendation 15. Processes for updating mHealth SRs should adapt to the dynamic mHealth app market.*

The apps market, where new apps are regularly introduced, existing apps are updated, and the features of apps change, can pose a challenge to updating mHealth SRs. Foreseeing updating processes that reflect this dynamic field is essential to ensure that SRs remain current, applicable, and relevant to the target populations. This update will allow the most effective mHealth interventions are identified and recommended to patients. Consider developing living systematic reviews, that is, systematic reviews which are “continually updated, incorporating relevant new evidence as it becomes available” [13]. Lessons learned during the COVID-19 pandemic for updating living SRs may be applicable to mHealth living SRs [14].

*Recommendation 16. Rapid reviews and evidence maps can provide helpful information in mHealth by offering a quicker evidence synthesis and research gap identification.*

Rapid reviews are systematic reviews with accelerated evidence synthesis processes, while evidence maps provide a visual summary of the available evidence on a particular topic. Both approaches are helpful in mHealth, where new technologies are rapidly emerging, and there is a need to keep up with the latest developments. Rapid reviews and evidence maps can help decision-makers, policymakers, and researchers prioritize areas for further investigation or action in mHealth and accelerate the translation of evidence into practice and policy. Follow recognised guidance for rapid reviews and evidence map development [15, 16].

**V. Recommendations that apply to several review stages**

*Recommendation 17. SRs should try to capture the complexity of mHealth interventions.*

mHealth interventions are complex interventions. Addressing this complexity in SRs is critical to evaluate the effectiveness of mHealth interventions. Factors contributing to the complexity of mHealth interventions include their integration with other health services, interactions between multiple technology components, user adherence and engagement, and contextual factors such as culture, society, and economics. Addressing complexity throughout the review process is essential for clarifying the review question and planning searches, data extraction, and analyses (quantitative or qualitative study)[17].

*Recommendation 18. Consider using a taxonomy of mHealth interventions to improve the clarity, organization, and evaluation of mHealth interventions in SRs*

A taxonomy is a systematic approach to organizing and categorizing information. mHealth interventions can take many forms, such as text messages supporting medication adherence, telemedicine consultations, and wearable devices tracking physical activity. Thus, a common taxonomy for mHealth interventions is essential for organizing and categorizing the interventions' components. Using a common taxonomy to classify and identify mHealth interventions would make it easier to apply the review inclusion criteria, determine which is the intervention and comparator in a study, establish meaningful comparisons, organize the information, evaluate the impact of different interventions, communicate more effectively with others, and identify gaps in the field. An example is the Cochrane Effective Practice and Organisation of Care (EPOC) taxonomy [18], which has been used by Cochrane authors in SRs of health systems interventions.

**Bibliography**

1. Peters MDJ, Marnie C, Tricco AC, Pollock D, Munn Z, Alexander L, et al. Updated Methodological Guidance for the Conduct of Scoping Reviews. JBI Evid Synth. 2020 Oct;18(10):2119-26. PMID: 33038124. doi: 10.11124/jbies-20-00167.

2. Nouri R, S RNK, Ghazisaeedi M, Marchand G, Yasini M. Criteria for Assessing the Quality of Mhealth Apps: A Systematic Review. J Am Med Inform Assoc. 2018 Aug 1;25(8):1089-98. PMID: 29788283. doi: 10.1093/jamia/ocy050.

3. Masterson Creber RM, Maurer MS, Reading M, Hiraldo G, Hickey KT, Iribarren S. Review and Nalysis of Existing Mobile Phone Apps to Support Heart Failure Symptom Monitoring and Self-Care Management Using the Mobile Application Rating Scale (Mars). JMIR Mhealth Uhealth. 2016;4(2):e74. PMID: 27302310. doi: <https://doi.org/10.2196/mhealth.5882>.

4. McKay FH, Slykerman S, Dunn M. The App Behavior Change Scale: Creation of a Scale to Assess the Potential of Apps to Promote Behavior Change. JMIR Mhealth Uhealth. 2019 Jan 25;7(1):e11130. PMID: 30681967. doi: 10.2196/11130.

5. Ayiku L, Hudson T, Glover S, Walsh N, Adams R, Deane J, et al. The Nice Medline and Embase (Ovid) Health Apps Search Filters: Development of Validated Filters to Retrieve Evidence About Health Apps. Int J Technol Assess Health Care. 2020 Oct 27;37:e16. PMID: 33107420. doi: 10.1017/s026646232000080x.

6. Glanville J, Bayliss S, Booth A, Dundar Y, Fernandes H, Fleeman ND, et al. So Many Filters, So Little Time: The Development of a Search Filter Appraisal Checklist. J Med Libr Assoc. 2008 Oct;96(4):356-61. PMID: 18974813. doi: 10.3163/1536-5050.96.4.011.

7. Rhon DI, Fritz JM, Kerns RD, McGeary DD, Coleman BC, Farrokhi S, et al. Tidier-Telehealth: Precision in Reporting of Telehealth Interventions Used in Clinical Trials - Unique Considerations for the Template for the Intervention Description and Replication (Tidier) Checklist. BMC Med Res Methodol. 2022 Jun 2;22(1):161. PMID: 35655144. doi: 10.1186/s12874-022-01640-7.

8. Eysenbach G. Consort-Ehealth: Improving and Standardizing Evaluation Reports of Web-Based and Mobile Health Interventions. J Med Internet Res. 2011 Dec 31;13(4):e126. PMID: 22209829. doi: 10.2196/jmir.1923.

9. Stoyanov SR, Hides L, Kavanagh DJ, Zelenko O, Tjondronegoro D, Mani M. Mobile App Rating Scale: A New Tool for Assessing the Quality of Health Mobile Apps. JMIR mHealth uHealth. 2015;3(1):e27. PMID: 25760773. doi: <https://doi.org/10.2196/mhealth.3422>.

10. Haddaway N WM. Predicter (Predicting Time Requirements for Evidence Reviews). 2023 [cited 2023 29 December]; Available from: <https://predicter.github.io/#>.

11. Lopez-Alcalde J, Yakoub N, Wolf M, Munder T, von Elm E, Flückiger C, et al. The Ripi-F (Reporting Integrity of Psychological Interventions Delivered Face-to-Face) Checklist Was Developed to Guide Reporting of Treatment Integrity in Face-to-Face Psychological Interventions. J Clin Epidemiol. 2022 Nov;151:65-74. PMID: 35926822. doi: 10.1016/j.jclinepi.2022.07.013.

12. Meyerowitz-Katz G, Ravi S, Arnolda L, Feng X, Maberly G, Astell-Burt T. Rates of Attrition and Dropout in App-Based Interventions for Chronic Disease: Systematic Review and Meta-Analysis. J Med Internet Res. 2020;22(9):e20283. PMID: 32990635. doi: <https://doi.org/10.2196/20283>.

13. Elliott JH, Synnot A, Turner T, Simmonds M, Akl EA, McDonald S, et al. Living Systematic Review: 1. Introduction-the Why, What, When, and How. J Clin Epidemiol. 2017 Nov;91:23-30. PMID: 28912002. doi: 10.1016/j.jclinepi.2017.08.010.

14. Heron L, Buitrago-Garcia D, Ipekci AM, Baumann R, Imeri H, Salanti G, et al. How to Update a Living Systematic Review and Keep It Alive During a Pandemic: A Practical Guide. Systematic Reviews. 2023 2023/09/02;12(1):156. doi: 10.1186/s13643-023-02325-y.

15. Garritty C, Gartlehner G, Nussbaumer-Streit B, King VJ, Hamel C, Kamel C, et al. Cochrane Rapid Reviews Methods Group Offers Evidence-Informed Guidance to Conduct Rapid Reviews. J Clin Epidemiol. 2021 Feb;130:13-22. PMID: 33068715. doi: 10.1016/j.jclinepi.2020.10.007.

16. White H, Albers B, Gaarder M, Kornør H, Littell J, Marshall Z, et al. Guidance for Producing a Campbell Evidence and Gap Map. Campbell Syst Rev. 2020 Dec;16(4):e1125. PMID: 37016607. doi: 10.1002/cl2.1125.

17. Petticrew M, Anderson L, Elder R, Grimshaw J, Hopkins D, Hahn R, et al. Complex Interventions and Their Implications for Systematic Reviews: A Pragmatic Approach. Journal of Clinical Epidemiology. 2013 2013/11/01/;66(11):1209-14. doi: <https://doi.org/10.1016/j.jclinepi.2013.06.004>.

18. Effective Practice and Organisation of Care (EPOC). Epoc Taxonomy. 2015 [cited 2023 29 December]; Available from: <https://doi.org/10.5281/zenodo.5105850>.
